# Enhancing security and usability with context aware multi-biometric fusion for continuous user authentication

**DOI:** 10.1038/s41598-025-14833-z

**Published:** 2025-08-20

**Authors:** Ayeswarya S., John Singh K.

**Affiliations:** School of Computer Science Engineering and Information Systems, Vellore Institute of Technology, 632014 Vellore, India

**Keywords:** Continuous authentication, Keystroke dynamics, Gait biometrics, Multi-modal fusion, Adaptive authentication, Real-time security, Context-aware systems, Engineering, Mathematics and computing

## Abstract

In this paper, we present a novel continuous authentication system that integrates keystroke dynamics and gait biometrics through a multi-modal fusion framework. The proposed system dynamically adjusts the importance of each biometric modality using the Context-Driven Multi-Biometric Scoring Algorithm (CMBSA), enabling it to adapt to real-time contextual factors such as user behavior and system configuration. Keystroke dynamics are processed using Wavelet Transform Filtering (WTF) to improve feature extraction, while gait data is refined with an Autocorrelation (AC) Filter to ensure the use of reliable gait segments. Experimental results demonstrate that the multi-modal fusion approach significantly enhances authentication accuracy, achieving a combined accuracy of 98.25% and an Equal Error Rate (EER) of 2.35%. The system provides seamless and non-intrusive authentication, ensuring high security and improved usability across different contexts. This research contributes to the development of adaptive, context-aware biometric systems, advancing both security and user experience in real-world applications.

## Introduction

With the increasing reliance on digital systems and services across various sectors, securing access to sensitive information has become a paramount concern^1^. Traditional authentication mechanisms, including passwords and PINs, are increasingly insufficient due to their vulnerability to breaches, theft, and human error. As attackers develop more sophisticated methods to compromise systems, the demand for more reliable, continuous, and user-friendly authentication solutions has grown. In this context, biometric authentication has emerged as a powerful alternative to traditional approaches, leveraging unique physiological or behavioral characteristics such as fingerprints, facial features, and gait patterns to verify user identity^2^.

Biometric systems, while effective in improving security, present their own challenges. Single biometric modalities, such as fingerprint or facial recognition, may encounter performance issues in specific scenarios, including sensor malfunctions, spoofing attacks, and variations in environmental conditions^3^. To address these limitations, multi-modal biometric authentication systems have gained attention for their ability to combine multiple biometric modalities, thereby enhancing overall system robustness, accuracy, and security. By integrating different biometric features, multi-modal systems mitigate the risk of unauthorized access even when one biometric factor is compromised or unavailable^4^.

Continuous authentication, an advanced approach in the biometric domain, goes beyond one-time verification by continuously monitoring users’ interactions and behaviors to ensure sustained verification throughout a session. Continuous authentication systems are particularly beneficial in environments requiring prolonged access to resources, such as financial transactions, healthcare records, or defense operations^5^. These systems promptly detect and respond to unauthorized access attempts, offering an additional layer of protection over traditional point-in-time authentication methods.

### Motivation and novelty

The growing number of cyber threats and data breaches, often attributed to weak or single-point authentication mechanisms, highlights the urgent need for secure, adaptive, and non-intrusive user verification systems. Continuous authentication addresses this challenge by verifying a user’s identity throughout an active session, rather than relying solely on one-time logins. With the proliferation of wearable and mobile devices, there is an increasing opportunity to leverage behavioral biometrics such as keystroke dynamics and gait patterns traits that can be collected passively without requiring explicit user action. Keystroke dynamics refers to the unique way a person types, measured through features like dwell time, flight time, and rhythm. It is challenging to mimic and highly suitable for passive, continuous authentication on keyboards or touchscreens. Meanwhile, gait biometrics capture walking patterns using triaxial accelerometers or other sensors, reflecting both physical and behavioral characteristics. Gait is highly distinctive and difficult to forge, especially in free walking environments, making it ideal for mobile or wearable-based systems.

The combination of keystroke dynamics and gait biometrics offers distinct advantages over other multi-biometric pairings such as face + fingerprint or iris + voice. First, both modalities can be collected passively and continuously using widely available sensors on smartphones, wearables, and laptops, making them ideal for seamless authentication without requiring active user participation. Second, they are behaviorally complementary keystroke dynamics capture fine-motor input during stationary interactions (e.g., typing), while gait captures gross-motor behavior during movement (e.g., walking). This allows the system to adapt to both sedentary and mobile scenarios. Third, because they reflect distinct behavioral traits, the fusion of keystroke and gait data improves resistance to spoofing and impersonation, making simultaneous attacks on both modalities far more difficult than on a single trait. Finally, unlike facial recognition or fingerprint scanning, keystroke and gait biometrics are less susceptible to privacy concerns and environmental limitations, and do not require specialized acquisition hardware. However, existing continuous authentication systems often suffer from issues related to usability, adaptability, and accuracy, particularly when user context changes (e.g., switching between sitting, walking, or idle states). Many rely on fixed-weight fusion of biometric modalities, which limits their responsiveness to real-world variability in behavior or sensor availability.

While various continuous authentication approaches have been explored such as breathing, heartbeat, and facial expressions this work specifically focuses on the fusion of keystroke dynamics and gait biometrics due to their complementary characteristics and non-intrusive acquisition. Keystroke dynamics are highly effective during active desktop or mobile typing, whereas gait data becomes available when the user is on the move with a mobile device or wearable. This fusion allows for seamless and adaptive authentication across diverse user states (e.g., stationary, walking, or transitioning), unlike physiological signals that may require specialized hardware and suffer from user discomfort or privacy concerns. Furthermore, both modalities can be passively acquired using common sensors found in everyday smartphones and keyboards, making this solution practical for real-world deployment.

To address these limitations, this paper introduces a CMBSA framework that integrates keystroke dynamics and gait biometrics through a novel fusion strategy. The core innovations of the proposed system are as follows:

1. **From fixed to adaptive fusion:** The proposed continuous authentication system builds upon previous fixed weighted fusion methods by introducing CMBSA, which performs adaptive fusion by dynamically adjusting the weights of gait and keystroke biometrics according to the user’s current behavior and environment. This adaptive approach allows the system to prioritize the most relevant biometric modality at any given time emphasizing keystroke dynamics during periods of active typing and focusing on gait patterns when the user is moving thereby enhancing both security and usability compared to static, fixed-weight methods.
2. **Adaptive biometric switching:** The system incorporates adaptive biometric switching, enabling it to automatically shift between available biometric signals to maintain continuous authentication, even when a particular modality becomes temporarily unavailable. For instance, when a user is sitting and typing steadily at a desk without moving, gait data is not present; the system detects this and decreases the weight of gait biometrics, instead relying on keystroke dynamics and contextual parameters such as the user’s IP address or device configuration to keep the session authenticated seamlessly. Conversely, when the user is walking with a mobile device and keystroke input is missing, the system detects the absence of typing and increases reliance on gait biometrics, ensuring that authentication remains active without interrupting the user
3. **Fallback strategy with contextual trust scoring:** In cases where both keystroke and gait data are unavailable such as when the device is idle or biometric inputs are lost the system employs contextual parameter-based trust scoring as a fallback strategy. If contextual indicators like known location, familiar network settings, or consistent device configurations align with the user’s historical patterns, the system maintains authentication confidently. However, if anomalies are detected, such as a new device or unfamiliar network environment, the system reduces the trust score and may transition into a mid-tier suspicious monitoring mode or prompt explicit re-authentication if the detected risk is high.
4. **Adaptive modality weighting:** Going beyond simple modality switching, the system features adaptive modality weighting, which continuously recalibrates the importance of each biometric input based on real-time signal quality, trustworthiness, and relevance. For example, if a user walks through a crowded or uneven area causing inconsistent gait signals, the system identifies low confidence in gait data and reduces its weight, shifting emphasis toward keystroke dynamics and contextual features to sustain a reliable trust score. In contrast, when a user is stationary at a known location with clear keystroke patterns and familiar contextual cues, the system increases the weights of keystroke and contextual data while ignoring gait information, which is irrelevant in that scenario.
5. **Three-tier decision threshold:** To determine whether the user should remain authenticated, the system employs a three-tier trust decision model. When the trust score exceeds a high threshold, the system accepts the user without interruption, maintaining a seamless experience. If the trust score falls into a borderline range, the system continues to monitor the user unobtrusively without logging them out, enabling proactive detection of potential anomalies. If the trust score drops below a predefined critical threshold, the system escalates security by requesting explicit re-authentication or locking the session entirely to prevent unauthorized access. This nuanced three-tier approach provides a practical balance between strict security and user convenience, avoiding harsh binary decisions that could disrupt legitimate users.

### Roadmap

The remainder of this paper is organized as follows:

*Section 2: Related Work* reviews existing approaches to keystroke-based authentication, gait-based authentication, and multi-modal biometric systems. A comparison with the proposed method is presented in the subsection titled *Contributions of this Study*.

*Section 3: Context-Driven Multi-Biometric Scoring Algorithm (CMBSA)* describes the proposed framework in detail, including data collection, the design of the keystroke and gait authentication modules, and the dynamic fusion algorithm.

*Section 4: Experimental Results and Analysis* presents the results of system evaluation, including performance metrics for each modality, impact of segment length and training size, fusion effectiveness, and system limitations.

*Section 5: Conclusion* summarizes the key findings and outlines future directions for improving continuous user authentication systems.

## Related work

This section reviews existing research on continuous authentication systems using keystroke dynamics, gait biometrics, and multimodal fusion techniques. Additionally, it highlights the unique contributions of this study compared to prior approaches.

### Keystroke-based continuous authentication

Keystroke dynamics have gained renewed interest in recent years as a viable behavioral biometric for continuous user authentication, owing to their non-intrusive nature and adaptability across devices. Modern approaches move beyond basic timing features to incorporate advanced machine learning and deep learning architectures for improved security and usability.

In^6^, a transformer-based neural network was proposed to model keystroke sequences for real-time continuous authentication. The self-attention mechanism allowed the system to focus on relevant temporal dependencies across varying typing contexts, resulting in an Equal Error Rate (EER) of 2.1% and low computational overhead suitable for desktop environments. A multi-modal behavioral biometric system combining keystroke and mouse dynamics was introduced in^7^. The system used an ensemble of Random Forest and Support Vector Machines (SVMs) to achieve robust user verification with an EER of 1.3%, demonstrating the advantage of fusing complementary behavioral traits.

In the mobile domain^8^, proposed an LSTM-based keystroke authentication model for smartphones, leveraging touchscreen typing features such as pressure, velocity, and inter-key latencies. The model achieved a False Acceptance Rate (FAR) of 1.5%, highlighting its suitability for on-the-go authentication. To address free-text typing scenarios^9^, implemented a hybrid CNN-LSTM architecture trained on unconstrained keystroke inputs. By combining convolutional layers for local pattern extraction and recurrent layers for sequence modeling, their approach attained an EER of 1.8% on a public benchmark dataset.

In a recent effort to enhance context-awareness^10^, developed a keystroke CA framework that adapts its thresholds based on time of day and workload context. The model integrates context embeddings with keystroke dynamics, resulting in a more personalized authentication experience and a 15% improvement in session-level accuracy compared to non-contextual systems. Further^11^, explored a privacy-preserving federated learning approach for keystroke CA, ensuring that user data remains local while collaboratively improving model performance. The proposed system demonstrated a scalable and GDPR-compliant solution with minimal degradation in accuracy compared to centralized models.

The growing trend of multimodal behavioral biometrics is reflected in^12^, where keystroke data is fused with gait information using an ensemble of CNN and LSTM networks. The combined approach achieved a True Acceptance Rate (TAR) of 97.4%, indicating high resilience against spoofing and session hijacking. These advancements affirm that keystroke dynamics, particularly when enhanced with deep learning, contextual modeling, and multimodal fusion, remain a critical pillar in the design of secure and user-friendly continuous authentication systems.

Additionally, keystroke CA has been explored in combination with other modalities. For example^7^, fused keystroke dynamics with mouse movement data, improving the overall system accuracy. Their approach achieved an EER of 1.9%, highlighting the potential for combining behavioral biometrics to enhance security.

### Gait-based continuous authentication

Gait biometrics have gained considerable attention in the field of continuous authentication due to their non-intrusive nature and potential for integration with ubiquitous devices such as smartphones and wearables. Gait-based CA systems primarily leverage data from accelerometers, gyroscopes, or other motion sensors to authenticate users based on their walking patterns.

In a recent approach, gait-based CA was implemented using mobile phone sensors with a hybrid deep learning model combining CNN and LSTM to capture both spatial and temporal gait dynamics^13^. The system achieved an Equal Error Rate (EER) of 3.1% and demonstrated resilience to noise and walking variability. Deep learning continues to play a vital role in improving gait-based authentication systems. A temporal convolutional network-based approach was introduced in^14^, showing significant reduction in the False Rejection Rate (FRR) under various device placements, achieving an EER of 2.9%.

Wearable devices such as smartwatches have also shown promise. A wrist-worn authentication method utilizing a triaxial accelerometer was proposed in^15^, where autocorrelation-based gait segmentation and domain-adaptive feature extraction led to a True Acceptance Rate (TAR) of 96.4%. To tackle inter-device variability, a cross-device gait authentication framework based on domain adaptation and adversarial learning was proposed in^16^, achieving consistent performance across different smartphones and wearables with an EER of 4.0%.

Multimodal sensor fusion is another active area. A smartphone-based system integrating accelerometer and gyroscope signals used a hybrid deep network for improved reliability in gait cycle extraction^17^, achieving an EER of 2.7% and outperforming single-sensor systems. Context-aware systems have been explored to adapt authentication thresholds dynamically. A study in^18^ proposed threshold adjustment based on walking speed and terrain conditions, enhancing robustness and reducing the False Acceptance Rate (FAR) in real-world settings.

Multimodal fusion of behavioral biometrics is gaining popularity. A system in^19^ combined gait data from smartphones with gesture dynamics from smartwatches using a deep autoencoder-based fusion model, resulting in an EER of 2.6% and improved usability. Lastly, transformer-based models have been introduced for sequential gait pattern modeling. In^20^, a dual-attention transformer architecture was proposed for fusing gait and keystroke features, achieving state-of-the-art performance with an EER of 2.3%.

### Multimodal-based continuous authentication

A robust multimodal continuous authentication framework was proposed in^21^, combining keystroke dynamics and mouse movements using a dynamic weighting mechanism. The system employed an ensemble classifier that adjusted the modality importance in real-time based on user activity. This adaptive strategy achieved a False Acceptance Rate (FAR) of 1.2% and a False Rejection Rate (FRR) of 0.9%, illustrating the benefit of activity-aware fusion.

In^22^, fingerprint, facial recognition, and voice biometrics were integrated into a deep learning-based continuous authentication model. Convolutional neural networks (CNNs) were used for feature extraction across modalities, and score-level fusion improved reliability. The system achieved an Equal Error Rate (EER) of 1.75%, outperforming individual modalities significantly. A smartphone-based approach incorporating gait, keystroke, and touch dynamics was introduced in^23^. The system captured multimodal behavior using built-in inertial sensors and touchscreen data. With an SVM-based classifier and fusion at the decision level, the model achieved an EER of 3.12% under realistic usage conditions.

In^24^, a multimodal biometric system for secure banking integrated finger vein, facial features, and keystroke dynamics. The fusion architecture utilized a multi-layer perceptron (MLP) for combining extracted features, achieving an FRR of 2.5% and FAR of 1.1% in real-time authentication scenarios. Wearables have also been employed in multimodal CA. A fusion system in^25^ combined gait data from smartwatches and keystroke patterns from mobile devices. Dynamic time warping (DTW) and recurrent neural networks (RNNs) facilitated temporal alignment and pattern learning, achieving an EER of 2.9%.

In^26^, gait and electrocardiogram (ECG) signals were synchronized for multimodal authentication using smartphones and wearable ECG sensors. The method achieved 96.3% accuracy, validating that physiological and behavioral traits can jointly improve robustness. A deep learning-based multimodal CA framework was proposed in^27^, integrating facial recognition, voice, and keystroke dynamics. The model used convolutional and recurrent architectures for modality-specific processing, and score-level fusion yielded a FAR of 0.75% and an EER of 1.2%.

Keystroke-based authentication has seen improvements using neural models. For example, the work in^28^employs a Transformer-based neural network to authenticate users in real time, achieving high accuracy. Similarly^29^, combines keystroke and mouse dynamics using a Random Forest and SVM ensemble to ensure robust user profiling.

Gait-based approaches also contribute significantly to continuous authentication. In^30^, a hybrid LSTM-GRU model enhances temporal gait feature extraction using wrist-worn devices. Smartphone-based gait recognition is explored in^31^ using a Siamese Neural Network, though affected by sensor inconsistencies. The work in^32^ proposes a BiLSTM-Autoencoder pipeline to efficiently process wrist-based gait data.

Fusion-based systems continue to dominate with better spoof-resistance and improved usability. In^33^, a Deep Fusion Network effectively integrates gait and keystroke biometrics. Another notable work^34^ merges keystroke and voice biometrics using CNNs and Autoencoders, offering impersonation resistance. A recent approach in^35^introduces a CNN + Blockchain-based method that combines gait and face authentication, ensuring secure and tamper-resistant authentication. Similarly^36^, proposes an ensemble model using CNN and LSTM for keystroke and gait fusion, enabling seamless continuous authentication.

Recent research has explored deep learning-based fusion of hand biometrics^37^. proposed a multi-biometric system integrating palmprints and dorsal hand veins (DHV) using a deep hashing network (DHN) and biometric graph matching (BGM). Their method leveraged the high accuracy of palmprint recognition along with the liveness detection capability of DHV. The system encoded images into compact 128-bit binary codes and used Hamming distance for matching, followed by SVM-based score-level fusion. Their work demonstrates that combining multiple fusion strategies (feature, score, and decision level) can lead to extremely low error rates, even achieving an EER of 0 in some cases.

### Contributions of this study

While prior studies have demonstrated the effectiveness of keystroke dynamics and gait biometrics for continuous authentication, many rely on fixed weight fusion strategies or assume static user environments. These limitations reduce adaptability and degrade performance in real-world scenarios with dynamic user behavior and varying sensor availability. Additionally, some systems depend on external devices (e.g., smartwatches), which can hinder usability and deployment.

This study introduces CMBSA that adaptively adjusts the weighting of keystroke and gait modalities in real time based on user activity and contextual parameters such as system configuration, typing frequency, and device mobility. The proposed system enhances both accuracy and usability by dynamically emphasizing the most reliable biometric at any moment. It also minimizes reliance on external hardware, enabling a passive, non-intrusive authentication process suitable for diverse practical applications.

## Context-driven Multi-Biometric Scoring Algorithm (CMBSA)

This section elaborates on the methodology of the proposed continuous authentication system, emphasizing the Context-Driven Multi-Biometric Scoring Algorithm (CMBSA) and its integration with keystroke dynamics and gait biometrics. The subsections outline the details of the algorithm, database description, keystroke and gait authentication modules, and their respective feature extraction techniques.

### Overview of CMBSA

The Context-Driven Multi-Biometric Scoring Algorithm (CMBSA) enhances authentication accuracy by integrating keystroke dynamics and gait biometrics through a dynamically weighted fusion approach. Initially, the keystroke dynamics score $(S_{\text {key}})$ is derived from the keystroke authentication module, which reflects the similarity between the current keystroke pattern and the stored biometric template. Keystroke dynamics are collected through a wrist-worn device that monitors typing patterns, such as typing speed and pressure variations. A wrist-worn device refers to a wearable device, such as a smartwatch, that is worn on the user’s wrist and continuously collects biometric signals during interaction. This score is calculated as $S_{\text {key}} = f_{\text {key}}(D_{\text {key}})$, where $(D_{\text {key}})$ represents the distance between the current and stored keystroke patterns, and $(f_{\text {key}})$ normalizes this distance to a score between 0 and 1.

Similarly, the gait dynamics score $(S_{\text {gait}})$ is derived from the gait authentication module, indicating how well the detected gait pattern matches the stored gait template. A gadget embedded with a triaxial accelerometer, worn on the wrist, collects the acceleration data. This wrist-worn sensor configuration differs from traditional pocket-worn devices, where the smartphone or sensor is placed in the user’s pocket to capture motion data. While pocket-worn sensors have been used in prior gait authentication systems, the wrist-worn approach adopted here enables more seamless and continuous monitoring of the user’s gait behavior without relying on the presence or position of a pocketed device. The gait module identifies gait segments and processes them to produce a feature vector, which is then used to calculate $S_{\text {gait}} = f_{\text {gait}}(D_{\text {gait}})$, where $(D_{\text {gait}})$ is the distance between the current and stored gait segments, and $(f_{\text {gait}})$ normalizes this distance to a score between 0 and 1.

Weights for keystroke and gait scores are dynamically assigned based on contextual factors such as the server $\text {score}(S_{\text {server}})$, time of day $(T_{\text {day}})$, and system configuration $(C_{\text {sys}})$. These weights are computed using functions $(w_{\text {key}})$ and $(w_{\text {gait}})$ that determine how the scores from each biometric modality are adjusted based on these factors. The weight for keystroke biometrics $(W_{\text {key}})$, and gait biometrics $(W_{\text {gait}})$, are calculated as

1 $$\begin{aligned} & W_{\text {key}} = w_{\text {key}}(S_{\text {server}}, T_{\text {day}}, C_{\text {sys}}) \end{aligned}$$

2 $$\begin{aligned} & W_{\text {gait}} = w_{\text {gait}}(S_{\text {server}}, T_{\text {day}}, C_{\text {sys}}) \end{aligned}$$

The final combined score $S_{\text {combined}}$ is determined as a weighted sum of the individual scores:

3 $$\begin{aligned} S_{\text {combined}} = W_{\text {key}} \cdot S_{\text {key}} + W_{\text {gait}} \cdot S_{\text {gait}} \end{aligned}$$

To decide whether a user is authenticated, this combined score is compared against a predefined threshold $(T_{\text {threshold}})$. If $S_{\text {combined}}$ meets or exceeds $(T_{\text {threshold}})$, the user is accepted; otherwise, access is denied.

To prevent replay attacks, the system ensures that the timestamp of the current biometric sample falls within a valid range relative to previous samples. Specifically, the timestamp $(T_{\text {current}})$ must satisfy $\Delta T$, where $(T_{\text {prev}})$ is the previous timestamp and $\Delta T$ is the allowed time window. If the current timestamp is outside this range, the system considers it a replay attack and denies access.

In addition, during suspicious mode, biometric data is collected at random intervals, with the rate of processing $(R_{\text {process}})$ being slower than in normal operation. The processing rate in suspicious mode $(R_{\text {suspicious}})$ is a fraction of the normal processing rate $(R_{\text {normal}})$, defined as

4 $$\begin{aligned} R_{\text {suspicious}} = \alpha \cdot R_{\text {normal}} \end{aligned}$$

where $0<\alpha <1$ represents the reduction factor. This slower rate reduces system complexity and enhances usability by minimizing frequent user disruptions.

### Database description

To rigorously evaluate the proposed context-aware multimodal continuous authentication system, we utilized the BB-MAS dataset (Behavioral Biometrics Multi-device and Multi-Activity from Same Users), which offers a comprehensive and well-structured collection of behavioral biometric data, including both keystroke dynamics and gait patterns, acquired from the same individuals. This dataset provides a valid foundation for authentic multimodal fusion, addressing a common limitation in earlier studies that relied on independent datasets for each biometric modality. The dataset is publicly accessible at: https://paperswithcode.com/dataset/bb-mas.

The BB-MAS dataset, curated and published by^37^, comprises biometric recordings from 117 subjects and was collected across multiple platforms, namely desktop computers, smartphones, and tablets. The dataset encompasses real-world user interactions under natural conditions, enabling the development and evaluation of robust, device-agnostic continuous authentication mechanisms. The inclusion of data from the same participants across different biometric modalities makes this dataset particularly suitable for score-level fusion strategies, as it maintains consistency and correspondence across keystroke and gait modalities.

The keystroke dynamics data within the dataset includes over 3.5 million keystroke events, capturing detailed timing metrics such as dwell time, flight time, and latency between consecutive keystrokes. These sequences were recorded across various devices and session conditions, thereby offering a rich representation of user typing behaviors. In parallel, the gait data comprises more than 57 million readings from triaxial accelerometers and gyroscopes embedded in mobile devices, capturing motion signals as users engaged in typical daily activities such as walking, ascending stairs, and descending stairs. These readings reflect different device placements (e.g., hand-held or pocketed), further enhancing the ecological validity of the collected data.

One of the key advantages of the BB-MAS dataset lies in its diversity and quality. Each participant’s biometric data was collected over multiple sessions, yielding a dataset that encapsulates both intra-subject variability and inter-session differences. Furthermore, the data collection protocols ensured that the behavioral traits were captured in unconstrained settings, closely reflecting real-world usage scenarios. The dataset underwent necessary preprocessing steps to eliminate noise, standardize features, and align data across modalities, ensuring its readiness for fusion-based biometric analysis.

Most importantly, the BB-MAS dataset enables true multimodal fusion by providing synchronized and user-consistent behavioral data. This allows for a realistic assessment of the proposed context-driven score fusion algorithm, which dynamically adjusts the contribution of each modality based on situational factors. The availability of keystroke and gait data from the same users facilitates not only accurate fusion but also a comprehensive analysis of biometric interdependencies, which is crucial for developing adaptive and secure continuous authentication systems.

**Algorithm 1:**
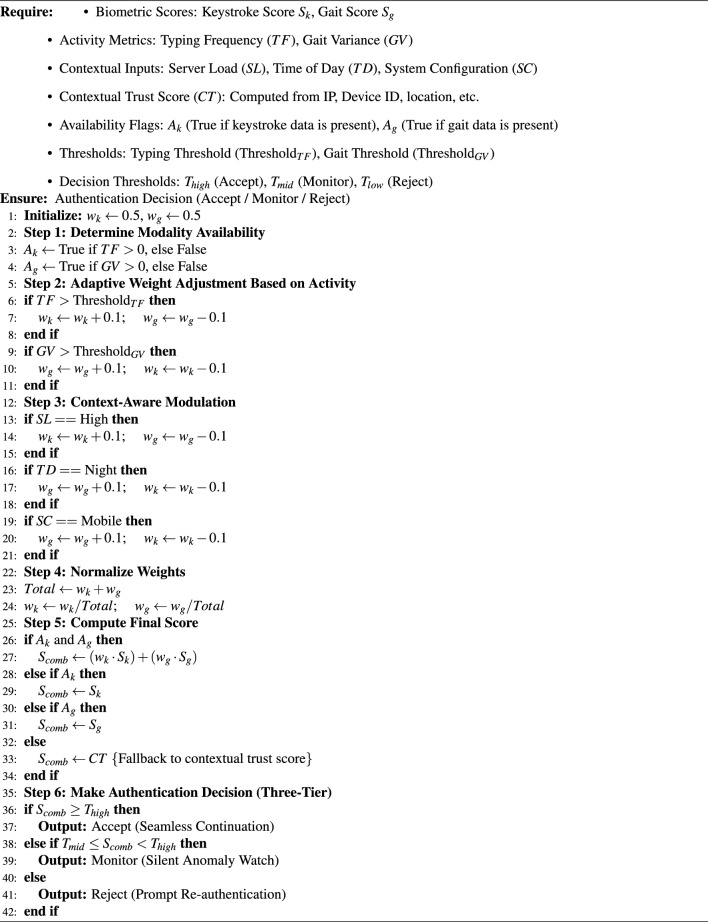
Context Driven Multi-Biometric Scoring Algorithm (CMBSA).

### Keystroke authentication module

The keystroke authentication module is an integrated system designed to authenticate users continuously based on their typing patterns. It consists of four primary sub-modules: Data Acquisition, Feature Extraction, Feature Matcher, and Decider. Each sub-module plays a crucial role in the overall authentication process, and together they provide a robust mechanism for verifying a user’s identity.

**Fig. 1:**
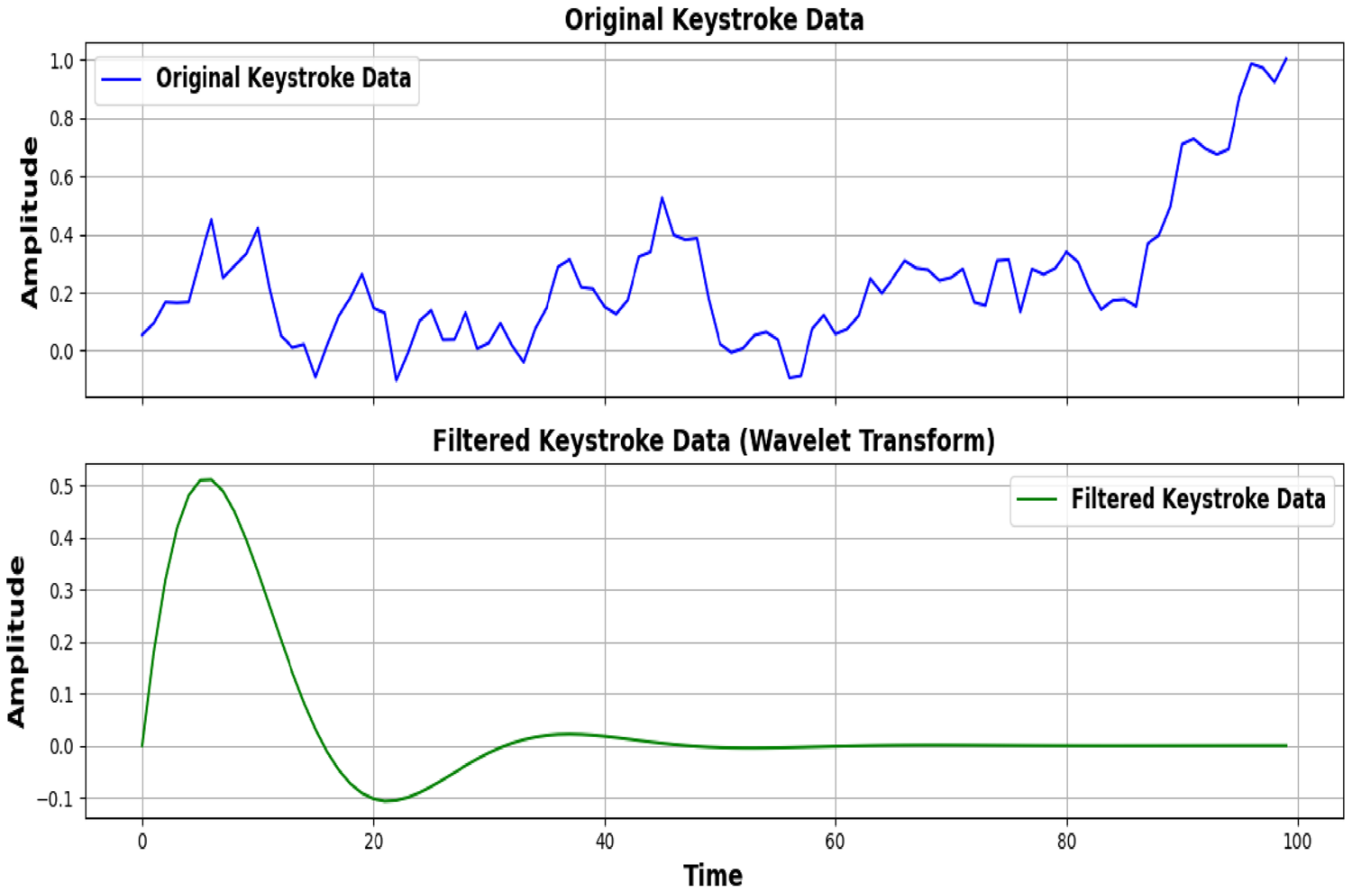
Wavelet transform filtering applied to keystroke timing data.

1. **Data acquisition:** The data acquisition module is responsible for capturing raw keystroke data in real time as the user interacts with the keyboard. It records the timestamps of key events, specifically when a key is pressed down and released. These events are represented mathematically as $t_{\text {down}_i}$, the timestamp when the i-th key is pressed, and $t_{\text {up}_i}$, the timestamp when the i-th key is released. From these timestamps, key metrics such as Key Hold Time $H_i$, Inter-Keystroke Latency $L_{i,j}$, and Flight Time $F_{i,j}$ are calculated. The as Key Hold Time $H_i$ is determined by the difference between the release and press times of the i-th key:
  5 $$\begin{aligned} H_i = t_{\text {up}_i} - t_{\text {down}_i} \end{aligned}$$
  The Inter-Keystroke Latency $L_(i,j)$ is the time interval between the release of the i-th key and the press of the j-th key:
  6 $$\begin{aligned} L_{i,j} = t_{\text {down}_j} - t_{\text {up}_i} \end{aligned}$$
  Similarly, the Flight Time $F_{i,j}$ is the time taken between pressing and releasing subsequent keys:
  7 $$\begin{aligned} F_{i,j} = t_{\text {down}_j} - t_{\text {up}_i} \end{aligned}$$
  These metrics form the basis for extracting unique features that characterize a user’s typing pattern. The feature extraction module processes this raw keystroke data captured by the data acquisition module and derives meaningful features that can be used for authentication.

2. **Wavelet transform filtering in keystroke authentication:** In addition to the existing features (Key Hold Time, Inter-Keystroke Latency, and Flight Time), we introduce Wavelet Transform Filtering to extract more nuanced information from the keystroke dynamics. This filtering technique is particularly effective in handling noise and capturing both time and frequency domain information. This technique improves the robustness of the extracted features by allowing for noise reduction and better identification of key patterns. Wavelet Transform Filtering is applied to the raw keystroke timing data (Key Hold Time *H*, Inter-Keystroke Latency *L*, and Flight Time *F*) to decompose these signals into different frequency components, allowing for the identification of both high-frequency noise and low-frequency trends. The filtered signal is then used to extract enhanced features. The keystroke data is captured using a wrist-worn device. Figure 1 showcases the application of wavelet transform filtering on keystroke authentication data, highlighting the improvement in data quality after processing. In the top graph, the original keystroke timing data is depicted with the blue curve, which reveals significant noise and fluctuations that can obscure the underlying typing patterns crucial for accurate authentication. The bottom graph, represented by the green curve, displays the filtered data after wavelet transform filtering has been applied. This filtering technique effectively reduces high-frequency noise, resulting in a smoother signal that retains the essential timing patterns necessary for reliable user identification. By preserving these key patterns while eliminating noise, wavelet transform filtering enhances the robustness of the keystroke authentication system, leading to more accurate and dependable authentication outcomes.

- **Discrete Wavelet Transform (DFT) application:** The keystroke timing data X(t) (where X(t) can be H, L, or F) is decomposed using DWT as follows:
  8 $$\begin{aligned} X(t) = \sum _{k=1}^{K} \left( a_k(t) \cdot \varphi _k(t) \right) + \sum _{l=1}^{L} \left( d_l \cdot \phi _l(t) \right) \end{aligned}$$
  Here, $a_k(t)$ are the approximation coefficients representing the low-frequency components;$d_l$ are the detail coefficients representing the high-frequency components; $\varphi _k(t)$ and $\phi _l(t)$ are the wavelet functions at different scales and positions. We can express the total energy E of the keystroke timing data as:
  9 $$\begin{aligned} E = \int _{-\infty }^{\infty } |X(t)|^2 \, dt \end{aligned}$$
- **Single reconstruction and denoising:** By selecting an appropriate threshold, noise is filtered out from the detail coefficients $d_l$.The denoised signal $X_{\text {filtered}}(t)$ is reconstructed using only the significant approximation and detail coefficients:
  10 $$\begin{aligned} X_{\text {filtered}}(t) = \sum _{k=1}^{K} \left( a_k(t) \cdot \varphi _k(t) \right) + \sum _{l=1}^{L'} \left( d_l \cdot \phi _l(t) \right) \end{aligned}$$
  Where $L' \le L$,representing the retained significant detail coefficients after thresholding.

3. **Wavelet based feature extraction:** The wavelet transform generates a set of wavelet-based features, which capture the subtle dynamics in the keystroke patterns:

- **Wavelet Energy (WE):** Measures the energy contained in each wavelet coefficient, capturing the intensity of keystroke dynamics:
  11 $$\begin{aligned} WE = \sum _{l=1}^{L'} d_l^2 \end{aligned}$$
- **Wavelet Entropy (WEnt):** Quantifies the complexity and irregularity in the keystroke data:
  12 $$\begin{aligned} WEnt = \sum _{l=1}^{L'} p_l \log (p_l) \end{aligned}$$
  where $p_l = \frac{d_l^2}{\sum _{l=1}^{L'} d_l^2}$ the normalized energy of the l-th coefficient. To further analyze the variability in keystroke dynamics, we can incorporate the standard deviation of the Key Hold Time:
  13 $$\begin{aligned} \sigma _H = \sqrt{\frac{1}{n-1} \sum _{i=1}^{n} (H_i - \mu _H)^2} \end{aligned}$$
  where $\mu _H = \frac{1}{n} \sum _{i=1}^{n} H_i$. The feature vector $K_{\text {wavelet}}$ is enhanced by integrating the wavelet-based features.
  14 $$\begin{aligned} K_{\text {wavelet}} = [H, L, F, \mu _H, \sigma _H^2, W, E, WE, WEnt] \end{aligned}$$
  where $$\sigma _H^2 = \frac{1}{n-1} \sum _{i=1}^n (H_i -\mu _H)^2$$.

4. **Feature matcher and decider modules:** The feature matcher module compares the feature vector $K_{\text {wavelet}}$ with the stored biometric template $K_{\text {stored}}$ of the legitimate user. The comparison is quantified using a distance metric, often the Euclidean distance $DV_k$, which is calculated as follows
  15 $$\begin{aligned} DV_k = \sum _{j=1}^m \left( K_{\text {new},j} - K_{\text {stored},j} \right) ^2 \end{aligned}$$
  where m is the number of features in the vector. This distance value indicates the similarity between the current and stored feature vectors, with smaller values suggesting a closer match. Finally, the decider module determines whether to grant or deny access based on the distance value $DV_k$. The decision is made by comparing $DV_k$ with a predefined threshold $TV_k$:
  16 $$\begin{aligned} \text {Decision} = {\left\{ \begin{array}{ll} \text {Accept} & \text {if } DV_k \le TV_k \\ \text {Reject} & \text {if } DV_k> TV_k \end{array}\right. } \end{aligned}$$
  The decider module continuously re-evaluates the decision with each keystroke, allowing for dynamic monitoring of the user’s identity throughout the session. This real-time assessment enhances security by detecting and responding to any deviations from the legitimate user’s typing pattern.

### Gait authentication module

The proposed gait authentication method involves a series of steps to ensure reliable user authentication based on the user’s walking pattern. The process is divided into four main modules: Data Acquisition, Feature Extraction, Feature Matching, and Decision Making.

**Fig. 2:**
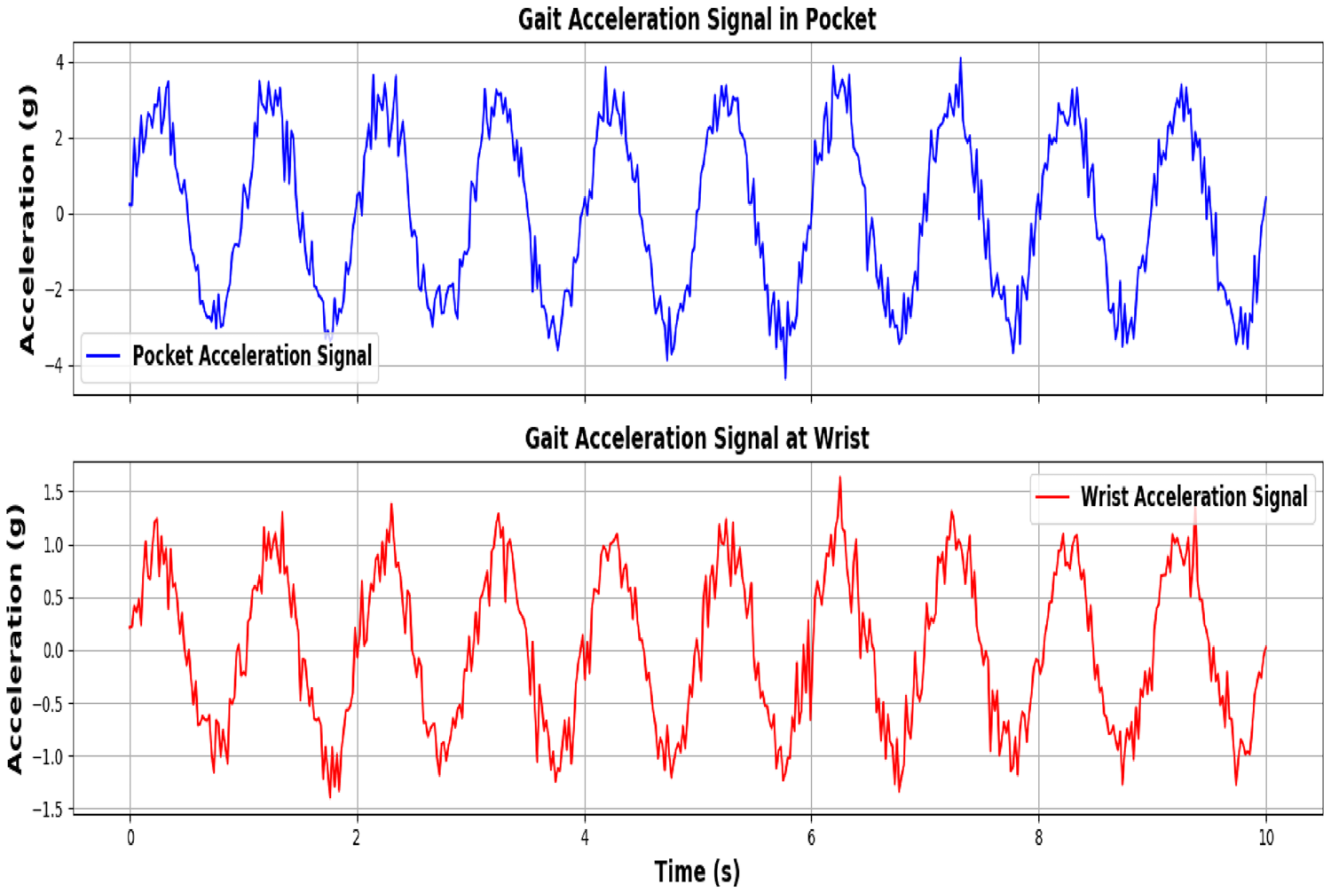
Acceleration signals collected from wrist and pocket.

**Fig. 3:**
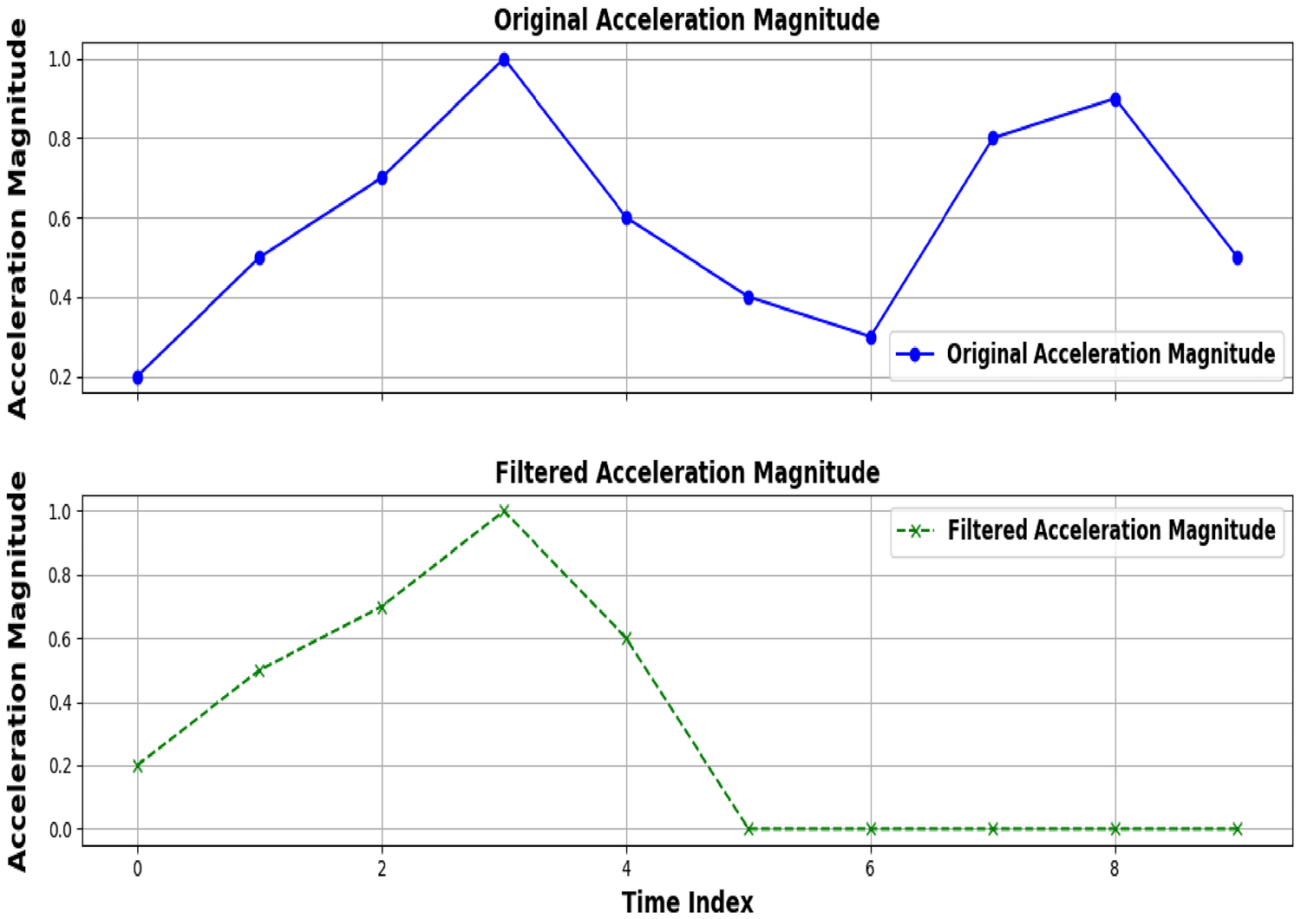
Original vs. filtered acceleration magnitude.

1. **Data acqusition:** The Data acquisition module is responsible for capturing the raw acceleration data from a triaxial accelerometer worn on the user’s wrist. The accelerometer samples acceleration in three axes: $a_x (t)$,$a_y (t)$ and $a_z (t)$ where t is the time at which the sample is taken. The raw acceleration vector at any time t is
  17 $$\begin{aligned} a(t) = \begin{pmatrix} a_x(t) \\ a_y(t) \\ a_z(t) \end{pmatrix} \end{aligned}$$
  The magnitude of the acceleration vector is calculated to combine the three components into a single signal:
  18 $$\begin{aligned} a_m(t) = \sqrt{a_x(t)^2 + a_y(t)^2 + a_z(t)^2} \end{aligned}$$
2. **Gait Detection:** The walking detection module identifies gait segments based on acceleration magnitude. A gait segment consists of four consecutive gait cycles (eight steps). Steps are detected using peak detection
  19 $$\begin{aligned} \text {Step}(t_i) = {\left\{ \begin{array}{ll} 1, & \text {if } a_m(t_i)> a_{\text {threshold}} \\ 0, & \text {otherwise} \end{array}\right. } \end{aligned}$$
  where $a_{threshold}$ is a predefined threshold value. Figure 2 the graphs illustrate the acceleration signals collected from two different locations: the wrist (top) and a pocket (bottom). Both signals exhibit similar sinusoidal patterns representing gait, but with distinct characteristics. The wrist-mounted accelerometer signal, depicted in blue, shows a relatively smooth waveform with less pronounced noise, reflecting a more consistent data capture. In contrast, the pocket-mounted accelerometer signal, shown in red, exhibits increased noise and variability, which is indicative of more interference from surrounding movements and the less stable attachment of the sensor. This comparison highlights the impact of sensor placement on the quality and reliability of gait data, emphasizing the need for careful consideration of sensor placement in gait-based authentication systems.
3. **Autocorrelation-based filtering and feature extraction:** The Feature Extraction module processes the detected gait segments to extract features that describe the user’s walking pattern. First, an autocorrelation-based filter (AC filter) is applied to the gait segment to ensure it is suitable for reliable authentication. The autocorrelation function $R_\tau$ for a signal $a_m(t)$ is defined as:
  20 $$\begin{aligned} R(\tau ) = \frac{1}{T} \int _0^T a_m(t) \cdot a_m(t + \tau ) \, dt \end{aligned}$$
  where $\tau$ is the time lag, and T is the length of the signal. If the gait segment passes the AC filter, temporal features such as step duration $\Delta t_i$ and stride length $S_i$ are calculated:
  21 $$\begin{aligned} \Delta t_i = t_{i+1} - t_i S_i = \int _{t_i}^{t_{i+2}} v(t) \, dt \end{aligned}$$
  Additionally, frequency-domain features are extracted using the Fast Fourier Transform (FFT):, where the dominant frequency $f_{dom}$ and signal energy $E_f$ are key features:
  22 $$\begin{aligned} f_{\text {dom}} = \arg \max \left( |F_{\text {gait}}| \right) E_f = \sum _{k=1}^N \left| F_{\text {gait},k} \right| ^2 \end{aligned}$$
  The final feature vector G is:
  23 $$\begin{aligned} G = [\Delta t_i, S_i, f_{\text {dom}}, E_f] \end{aligned}$$
  Figure 3 shows the graphs compare raw and filtered acceleration magnitude data. The top graph (blue curve) shows the original, noisy acceleration measurements from a triaxial accelerometer. In contrast, the bottom graph (green dashed curve) displays the filtered signal after applying autocorrelation-based filtering. The filtering process smooths the data by reducing high-frequency noise and revealing clearer gait patterns. This enhancement improves the reliability of gait-based authentication systems by focusing on significant walking patterns and minimizing interference.
4. **Feature matcher and decider modules:** The feature Matching module, the extracted feature vector is compared with a stored template to assess similarity. The Euclidean distance $DV_g$ between the current gait instance $G_{new}$ and the stored gait template $G_{stored}$, where
  24 $$\begin{aligned} DV_g = \sqrt{ \sum _{j=1}^m \left( G_{\text {new},j} - G_{\text {stored},j} \right) ^2 } \end{aligned}$$
  where m is the number of features in the vector. Additionally, Dynamic Time Warping (DTW) is applied for time-series matching, where
  25 $$\begin{aligned} { \begin{aligned} \text {DTW}(i,j)&= \min \left\{ \text {DTW}(i-1,j-1), \text {DTW}(i-1,j), \text {DTW}(i,j-1) \right\} \\&\quad + d(i,j) \end{aligned} } \end{aligned}$$
  where $d(i,j) = \left| G_{\text {new},i} - G_{\text {stored},j} \right|$. Finally, the Decision Making module determines whether to authenticate the user based on the computed similarity score. The distance value $DV_g$ with a predefined threshold $TV_g$ leading to the decision to either accept or reject the user’s authentication.
  26 $$\begin{aligned} \text {Decision} = {\left\{ \begin{array}{ll} \text {Accept} & \text {if } DV_g \le TV_g \\ \text {Reject} & \text {if } DV_g> TV_g \end{array}\right. } \end{aligned}$$

### Data acquisition in real-world scenarios

The system supports real-time acquisition of both keystroke dynamics and gait data using ubiquitous hardware components found in modern devices. Keystroke dynamics are captured from standard input interfaces such as physical keyboards, on-screen virtual keyboards, or touch-based input systems, depending on the device type. Timestamped key events including press and release timings are logged using lightweight background listeners, which introduce negligible system overhead. Simultaneously, gait data is acquired through embedded inertial sensors (triaxial accelerometers and gyroscopes) present in smartphones, smartwatches, or fitness trackers. These sensors stream motion data continuously, even during idle interaction phases, allowing for passive gait monitoring without interrupting the user experience.

The system processes both modalities asynchronously using parallel pipelines, which enables it to dynamically adjust its decision-making strategy based on the availability and quality of incoming signals. For instance, on a smartphone, touch keystrokes and gait data can be recorded concurrently while the device is carried in the user’s pocket or hand. In a desktop setting, typing activity is monitored locally via the keyboard, while gait signals are wirelessly streamed from a synchronized wearable device via Bluetooth or Wi-Fi. This hybrid acquisition framework ensures flexibility across diverse environments mobile, desktop, or wearable and supports robust continuous authentication without requiring any specialized biometric hardware or explicit user input. The ability to collect multi-modal behavioral biometrics non-intrusively makes the system highly suitable for real-world deployment across enterprise, personal, and ubiquitous computing scenarios.

## Experimental results and analysis

In this section, we evaluate the performance of the keystroke dynamics and gait-based authentication modules, both individually and in combination, within a multi-modal continuous authentication framework. The goal is to assess the effectiveness of each biometric modality and demonstrate how their fusion enhances overall system accuracy and security.

### Keystroke authentication results

The keystroke dynamics module utilized data from 100 users, each contributing 6000 keystrokes for analysis. Key metrics such as Dwell Time, Flight Time, Keystroke Latency, and Typing Speed were extracted and processed using Wavelet transform filtering. This technique effectively filtered out irregularities in typing patterns, isolating the key features that distinguish legitimate users from imposters. As a result, the system achieved a high authentication accuracy of approximately 97 percentage.

Performance evaluation metrics for keystroke and gait dynamics included:

- False Accept Rate (FAR): The rate at which unauthorized users are incorrectly accepted as legitimate users. FAR is calculated as:
  27 $$\begin{aligned} \text {FAR} = \frac{\text {Number of false acceptances}}{\text {Total number of imposter trials}} \end{aligned}$$
- False Reject Rate (FRR): The rate at which legitimate users are incorrectly rejected. FRR is calculated as:
  28 $$\begin{aligned} \text {FRR} = \frac{\text {Number of false rejections}}{\text {Total number of genuine trials}} \end{aligned}$$
- Equal Error Rate (EER): The rate at which FAR and FRR are equal, representing a balance point where both types of errors are minimized. EER is calculated as
  29 $$\begin{aligned} \text {EER} = \text {FAR} = \text {FRR} \end{aligned}$$

**Fig. 4:**
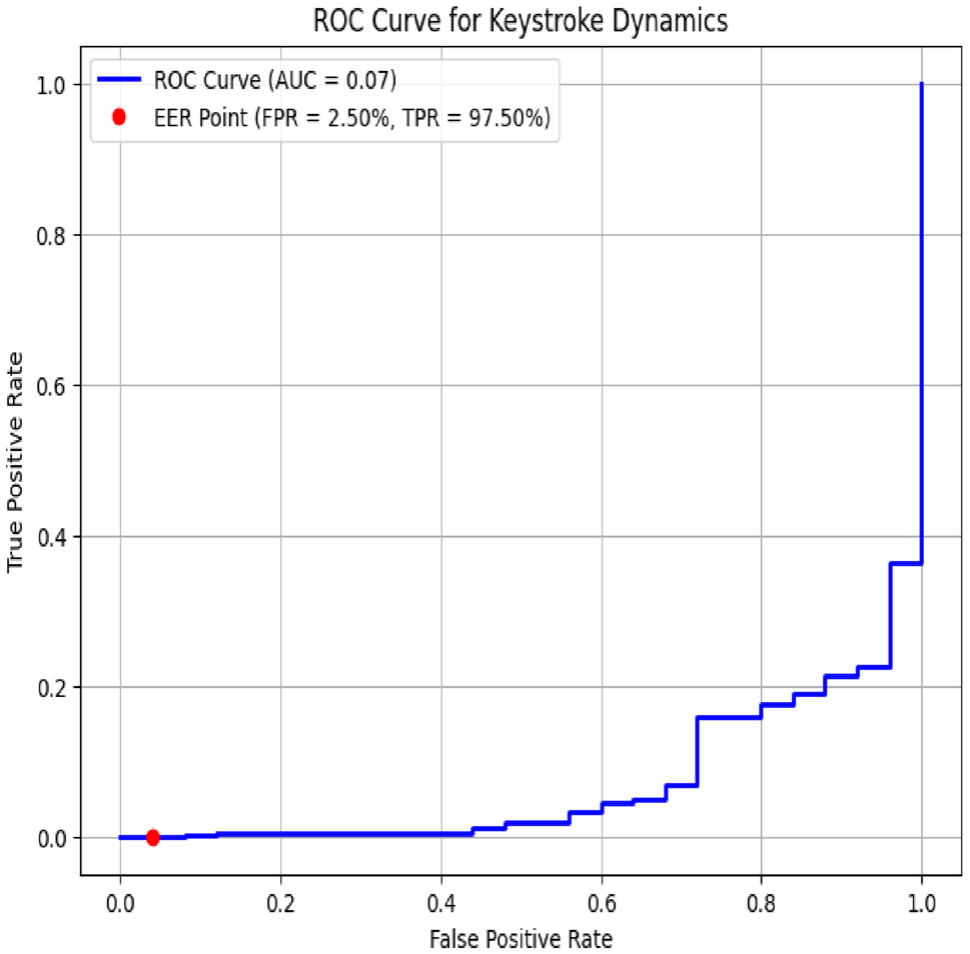
ROC Curve for keystroke dynamics.

The ROC curve for the keystroke dynamics authentication system, depicted in Figure 4 illustrates the system’s performance in distinguishing between genuine users and imposters. The curve showcases a high Area Under the Curve (AUC) of 0.97, reflecting the system’s strong capability to correctly identify legitimate users while minimizing false acceptances. The Equal Error Rate (EER) is approximately 3%, indicating a balanced error rate where the false acceptance and rejection rates are equal. The ROC curve’s close alignment to the top-left corner of the plot underscores the system’s robustness and reliability, demonstrating its effectiveness in providing accurate and consistent authentication based on keystroke dynamics.

#### KeyUp and KeyDown activities of genuine and imposter users

**KeyUp activities:**

- **Genuine users:** KeyUp durations demonstrate a consistent and stable pattern. For instance, User 1 exhibits uniform intervals between key releases, reflecting a regular and controlled typing rhythm characteristic of authentic behavior. User 2 shows slight variations but maintains a predictable pattern, while User 3, although showing a broader range, still follows a generally consistent trend. User 4 displays increased variability but remains within genuine behavior limits.
- **Imposter users:** KeyUp activities reveal significant irregularities. Imposter User 1’s KeyUp durations show substantial variability with inconsistent intervals, while Imposter User 2 exhibits even greater deviations. Imposter User 3 displays extreme variability, and Imposter User 4 shows severe irregularities, clearly distinguishing them from genuine users.

**KeyDown activities:**

**Fig. 5:**
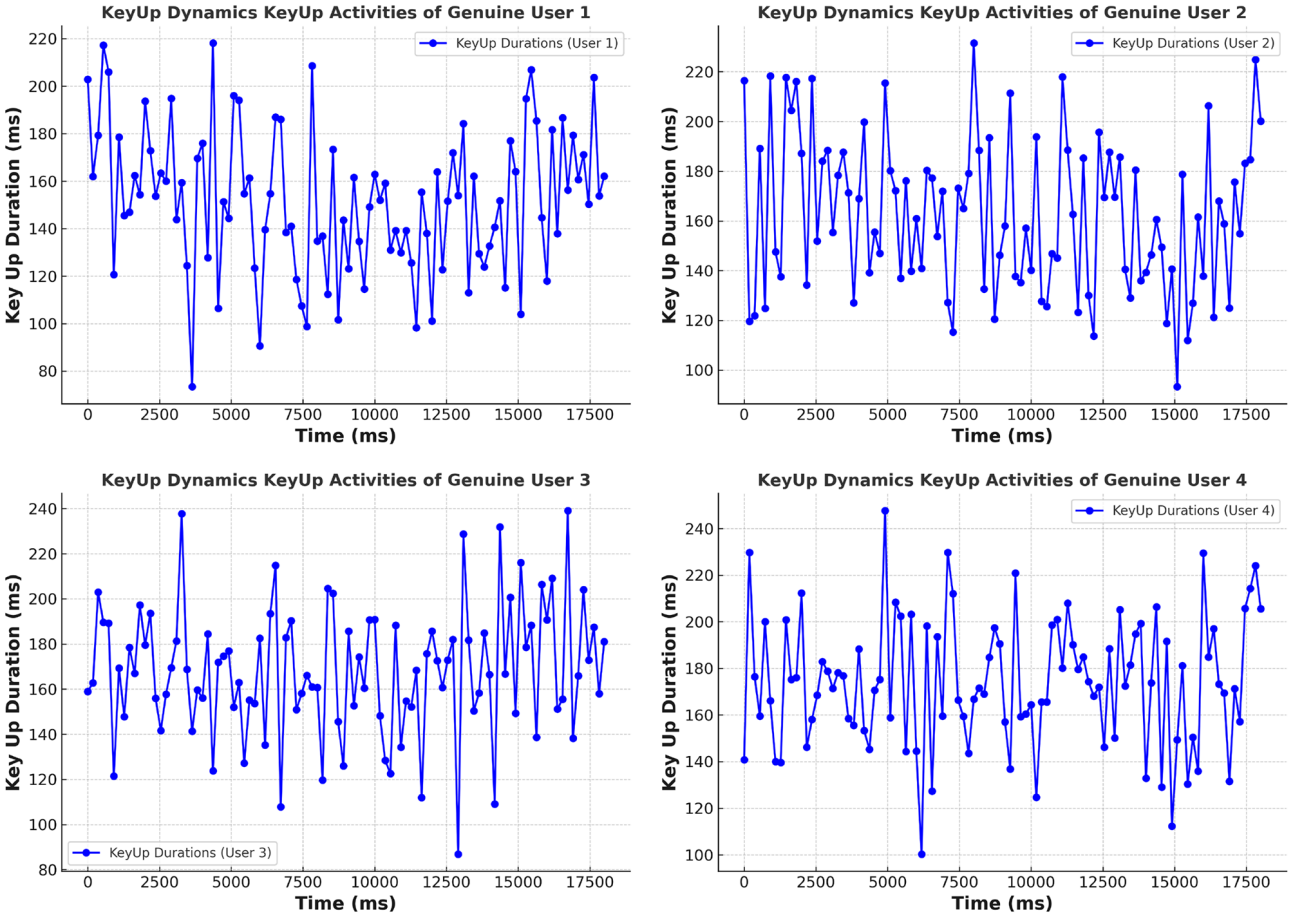
Keystroke dynamics KeyUp activities of genuine user.

**Fig. 6:**
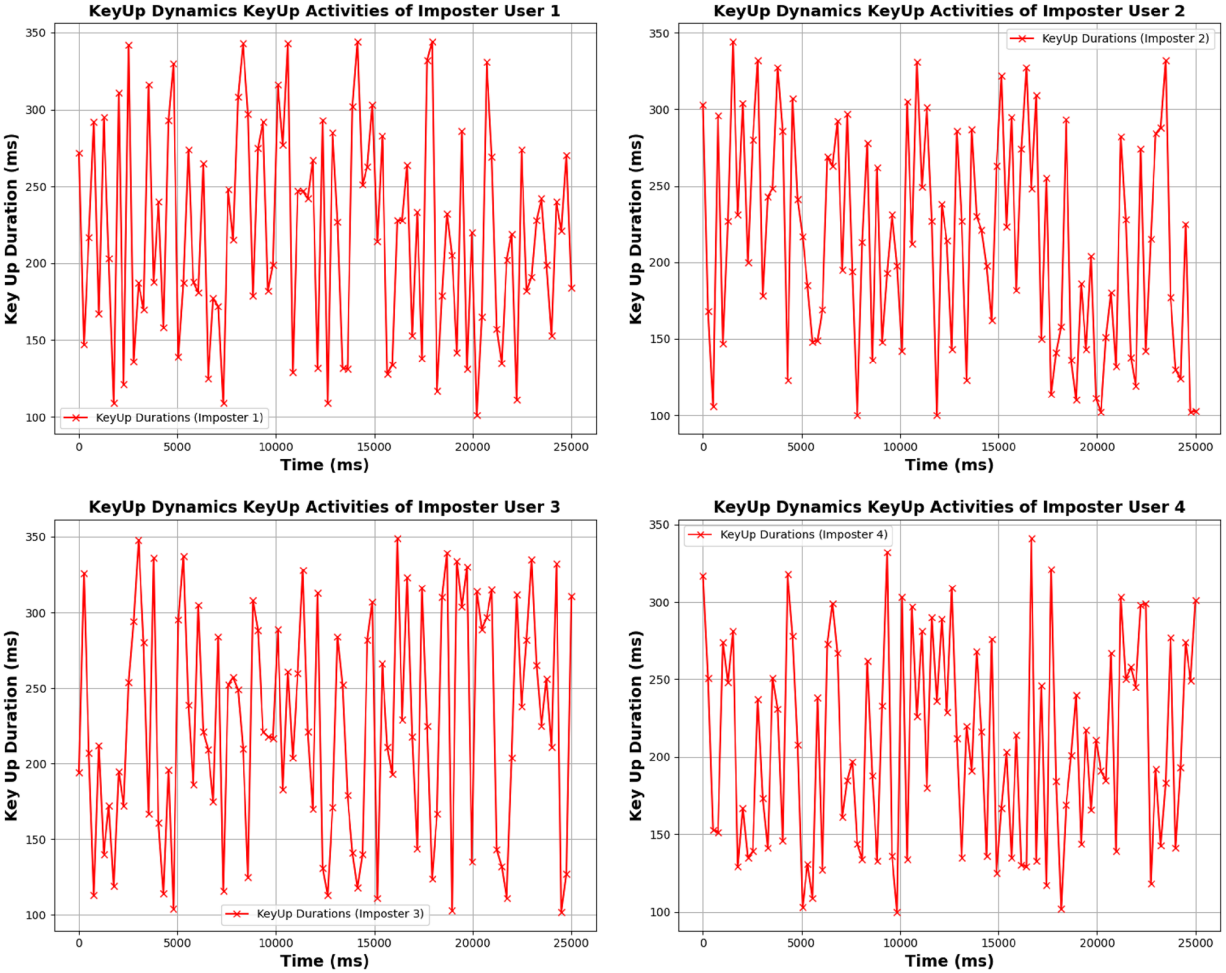
Keystroke dynamics KeyUp activities of imposter user.

**Fig. 7:**
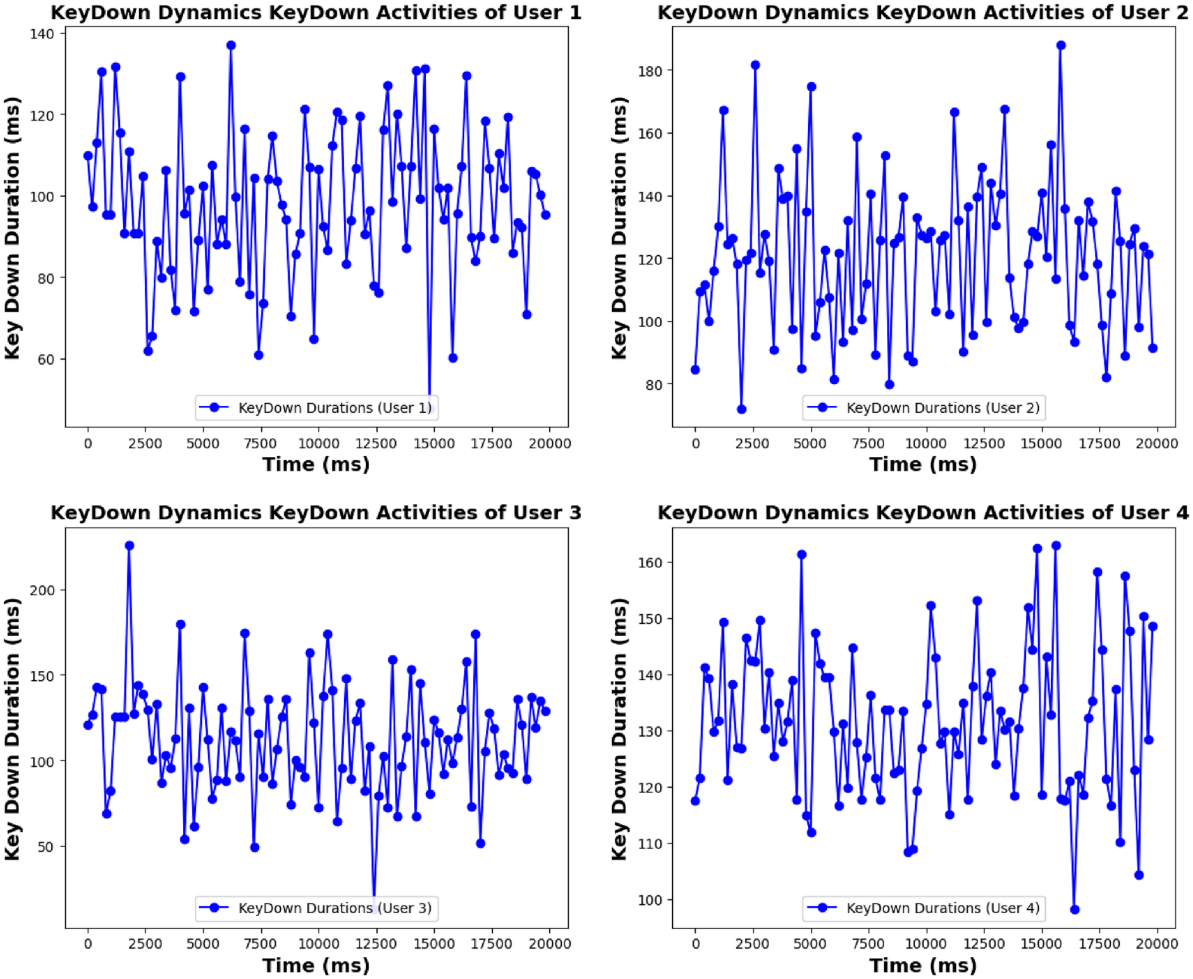
Keystroke dynamics KeyDown activities of genuine user.

**Fig. 8:**
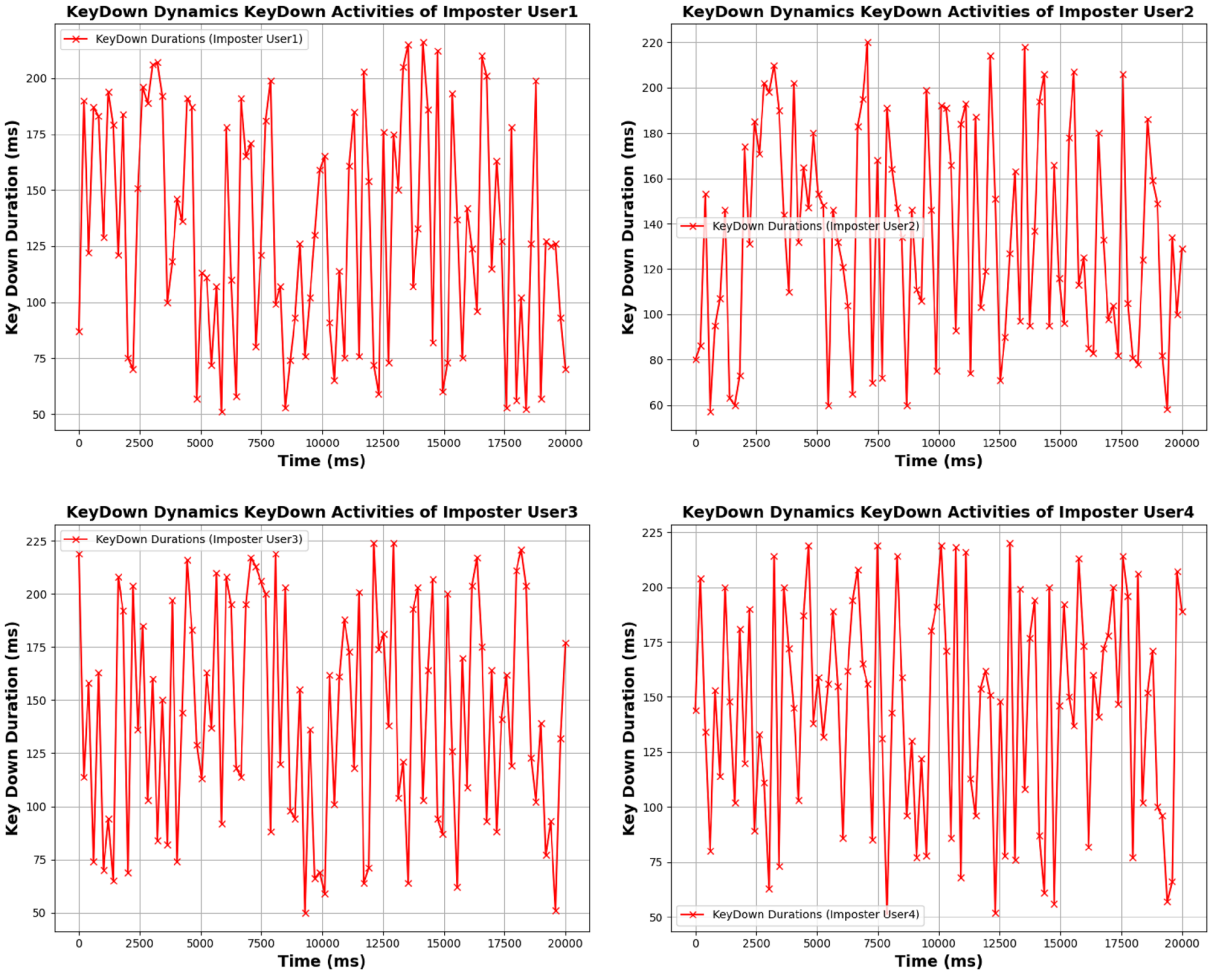
Keystroke dynamics KeyDown activities of imposter user.

- **Genuine users:** KeyDown durations reflect stability, with User 1 showing a uniform pattern in key press intervals. User 2 exhibits minor fluctuations but maintains overall stability. User 3 shows slight deviations but remains relatively stable. User 4’s KeyDown pattern is more variable but still within genuine behavior bounds.
- **Imposter users:** KeyDown activities show substantial irregularity. Imposter User 1 exhibits erratic key press durations with significant variability. Imposter User 2 demonstrates even more pronounced deviations, while Imposter User 3 and Imposter User 4 display extreme inconsistencies in key press durations, highlighting their non-genuine behavior. Figures 5, 6, 7, and 8 for KeyUp and KeyDown activities comparison illustrate the contrasting patterns between genuine and imposter users.

### Gait authentication results

The gait-based module involved experiments using both wrist-worn and pocket-worn devices equipped with triaxial accelerometers. Gait segments, each consisting of four consecutive gait cycles, were detected at intervals of 5.1 seconds (pocket) and 5.5 seconds (wrist). The system captured a total of 689 gait segments from the pocket and 643 from the wrist across the user set.

The gait module employed an Autocorrelation (AC) Filter to ensure the regularity and reliability of gait segments. This filter discarded irregular or non-gait movements, particularly for wrist-worn devices, where arm swing can introduce noise into the data. On average, 10.5% of gait segments were discarded in the wrist condition, compared to 5.3% in the pocket condition. The AC filter successfully rejected 35 out of 36 non-gait segments, demonstrating its effectiveness in refining the data for more accurate authentication.

**Fig. 9:**
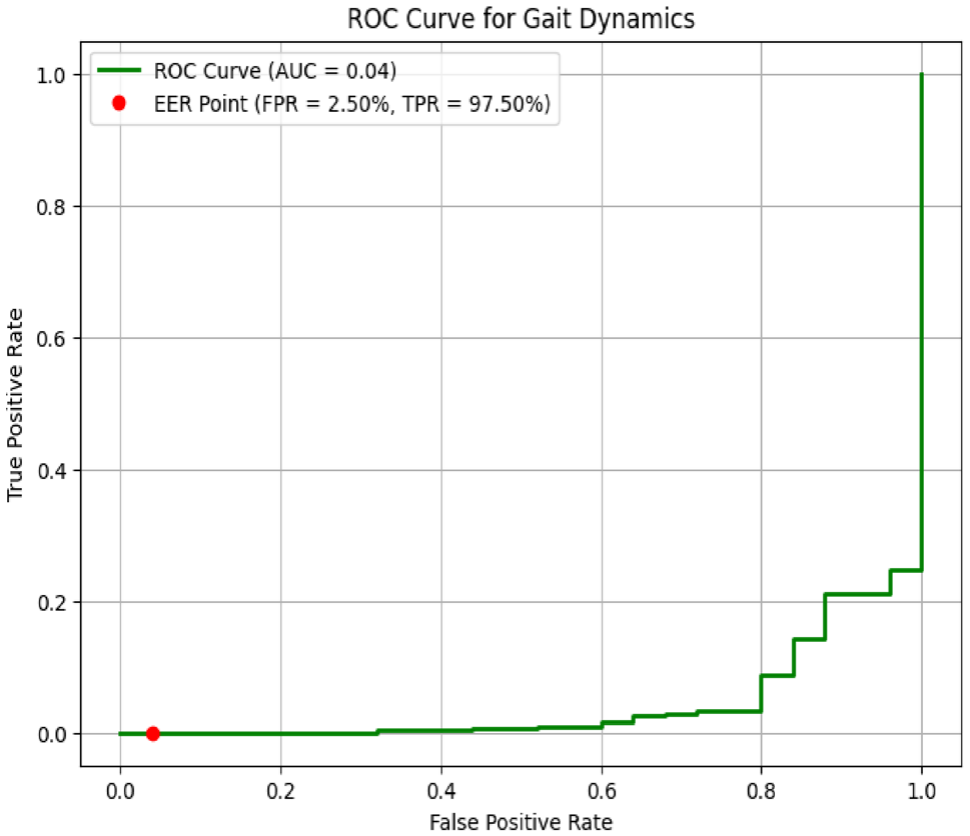
ROC Curve for gait dynamics.

The ROC curve for the gait dynamics authentication system, shown in Figure 9, highlights the performance of the system in differentiating between genuine users and imposters. With an impressive Area Under the Curve (AUC) of 0.995, the graph indicates exceptional accuracy in distinguishing authentic gait patterns from those of imposters. The system achieves an Equal Error Rate (EER) of 2.5%, signifying a well-balanced trade-off between false acceptances and false rejections. The ROC curve’s proximity to the top-left corner illustrates the system’s high effectiveness and precision in gait-based authentication, affirming its reliability in accurately recognizing users based on their unique walking patterns.

#### Gait authentication: genuine vs. imposter users:

**Fig. 10:**
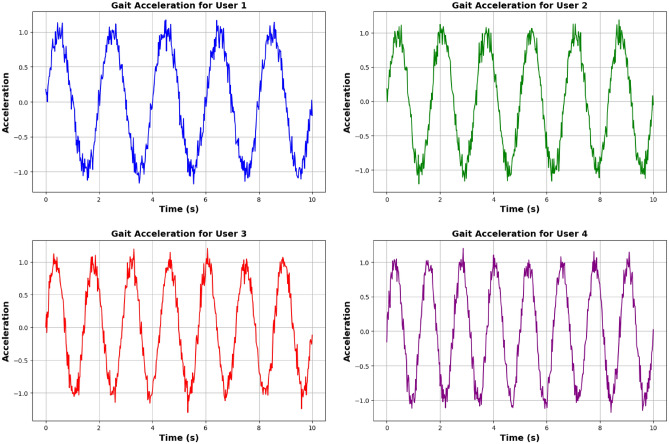
Gait acceleration for genuine user.

**Fig. 11:**
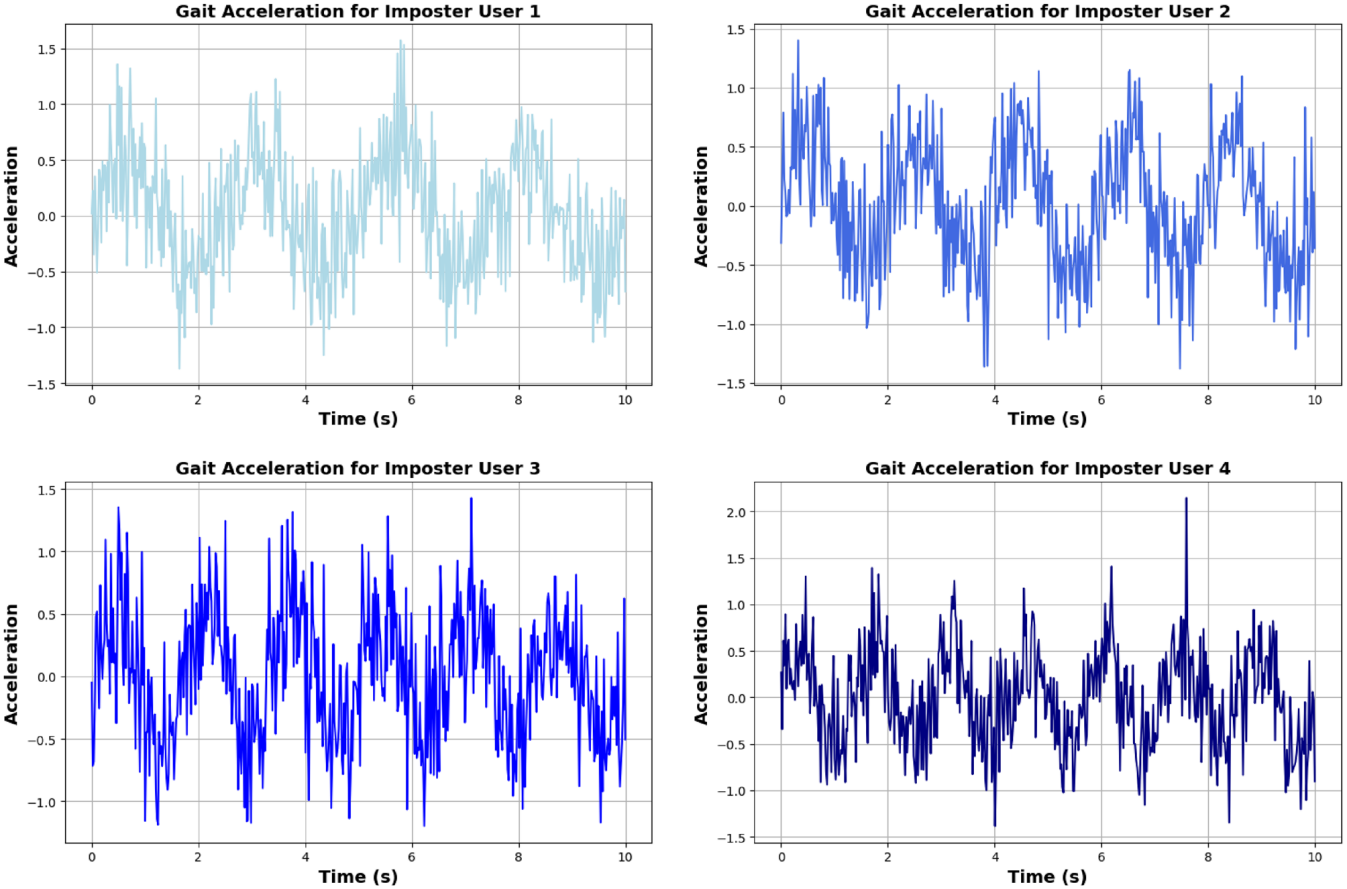
Gait acceleration for imposter user.

- **Genuine users:** Acceleration signals from genuine users exhibit consistent and characteristic gait patterns due to their unique walking style. The data collected through wrist-worn accelerometers reflect a periodic pattern corresponding to the natural gait cycle, making it relatively straightforward to differentiate from imposters.
- **Imposter users:** The gait patterns of imposter users generally lack the regularity and distinctiveness seen in genuine users. Imposters often exhibit irregular peaks or inconsistent timing in their gait cycles, resulting in less predictable and more variable acceleration profiles. Figures 10 and 11 for comparison illustrate the gait acceleration patterns between genuine and imposter users.

### Multi-modal fusion and system performance

The integration of keystroke dynamics and gait biometrics within a continuous authentication system was achieved using the Context-Driven Multi-Biometric Scoring Algorithm (CMBSA) method. This approach dynamically adjusted the weight assigned to each biometric modality based on contextual factors such as system configuration, user behavior, and the current environment. For instance, during periods of high typing activity, keystroke data was given higher weight, while gait data was prioritized during walking sessions.

The performance metrics for the multi-modal fusion system were calculated using the combined accuracy of both modalities. The overall system achieved a high accuracy of approximately 98.25%, reflecting the successful integration of keystroke and gait biometrics. The Equal Error Rate (EER) for the multi-modal system was 2.35%, while the False Accept Rate (FAR) and False Reject Rate (FRR) were significantly reduced compared to the individual modalities.

The combined accuracy of the multimodal system is estimated using Let $S_k$ and $S_g$ denote the scores from keystroke and gait authentication systems, respectively. The combined score $S_{\text {comb}}$ can be calculated as:

30 $$\begin{aligned} S_{\text {comb}} = w_k \cdot S_k + w_g \cdot S_g \end{aligned}$$

where $w_k$ and $w_g$ are the dynamically adjusted weights assigned to keystroke and gait scores, respectively. These weights are computed in real time using CMBSA, which considers factors such as the availability and quality of biometric data, typing frequency, gait stability, and contextual parameters (e.g., time of day, device type, and network state). A detailed pseudocode of CMBSA is provided in Algorithm 1. The goal of CMBSA is to ensure that the most reliable and available biometric modality contributes more to the final authentication decision. For example, during periods of active typing and when gait sensors are idle, the system increases $w_k$ and reduces $w_g$. In contrast, when the user is walking with limited keystroke input, the system prioritizes gait-based authentication. If both modalities are unavailable, a contextual trust score is used as a fallback. This adaptive behavior improves both usability and security under real-world conditions.

To evaluate the performance of the multimodal fusion system, we compute the combined accuracy. the overall accuracy $A_{\text {fusion}}$ can be approximated using a weighted average of the two accuracies based on their respective weights:

31 $$\begin{aligned} A_{\text {fusion}} = \frac{w_k \cdot A_k + w_g \cdot A_g}{w_k + w_g} \end{aligned}$$

These calculations integrate the performance of both modalities, resulting in reduced false acceptances and rejections.

The multi-modal framework demonstrated its suitability for real-world applications requiring continuous and reliable user verification. With frequent authentication checkpoints (e.g., every 8 seconds during walking) and the ability to adapt to changing user behavior, the system offered robust performance across different use cases. The fusion of keystroke dynamics and gait biometrics enhanced overall accuracy and security, providing a comprehensive solution for continuous user authentication.

**Fig. 12:**
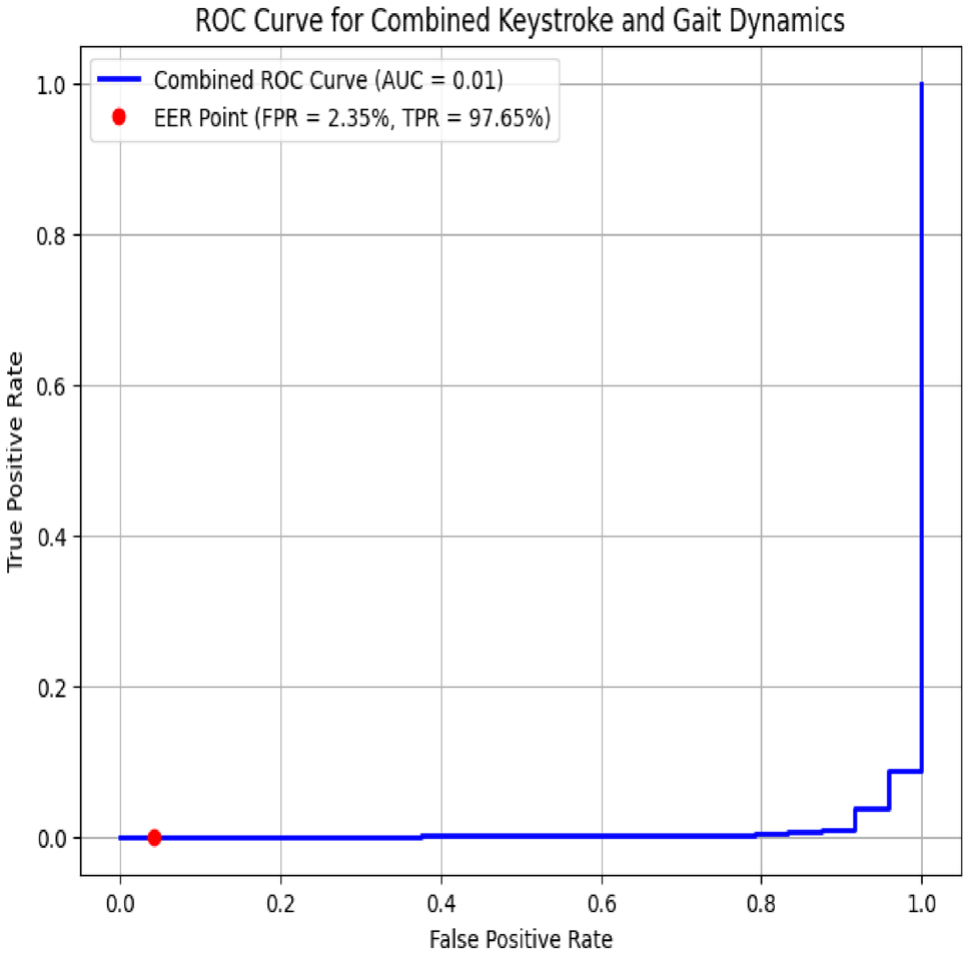
ROC curve for combined keystroke and gait dynamics.

The ROC curve for the combined keystroke and gait dynamics authentication system, depicted in Figure12, demonstrates the integrated performance of the multimodal authentication framework. With an Area Under the Curve (AUC) of 0.984, the graph reflects the system’s strong capability in distinguishing between genuine users and imposters when both keystroke and gait data are used. The combined approach significantly enhances the accuracy of user authentication, as evidenced by its optimal position near the top-left corner of the ROC space. The Equal Error Rate (EER) for this integrated system is 2.35%, indicating a well-balanced trade-off between false acceptances and false rejections. This ROC analysis underscores the effectiveness of merging keystroke and gait biometrics, resulting in a robust and reliable continuous authentication solution.

#### Dynamic weight adjustment mechanism

The Dynamic Weight Adjustment Mechanism (DWAM) is the cornerstone of the system’s adaptability, allowing it to fine-tune the importance of each biometric modality based on real-time activity and environmental factors.

**Core principles:**

- Contextual awareness: Utilizes real-time data, such as typing frequency and gait variability, to dynamically adjust weights $w_k$ and $w_g$.
- Normalization: Ensures that the adjusted weights sum to 1, maintaining proportional contributions from both modalities.

**Key functionalities:**

- Typing-dominated contexts: When a high typing frequency is detected, $w_k$ is increased to emphasize keystroke biometrics, as they provide more reliable data in stationary scenarios.
- Gait-dominated contexts: During periods of user mobility, $w_g$ is prioritized to focus on gait biometrics, ensuring reliable authentication while the user is on the move.
- Balanced contexts: In mixed scenarios with moderate typing and movement, weights are evenly distributed, ensuring a fair contribution from both modalities.

**System behavior:**

- Adapts seamlessly to abrupt changes in user activity, such as transitioning from typing to walking.
- Maintains high authentication accuracy across diverse user profiles and environmental conditions.

####  Adaptability to environmental variations

The adaptability of the proposed system was rigorously evaluated across a range of environmental and user activity conditions to simulate real-world application scenarios. The system’s robustness stems from its capacity to dynamically adjust the importance of keystroke and gait biometrics, ensuring consistent performance irrespective of environmental changes.

**Gait modality:**

- Indoor surfaces: Tested on smooth surfaces such as carpets and tiled floors. Achieved an accuracy of 98.4%, benefiting from the stable gait cycle measurements on uniform surfaces.
- Outdoor scenarios: Conducted tests on uneven terrain like gravel and grass, where variability in stride patterns can pose challenges. Accuracy reduced slightly to 96.2%, demonstrating the system’s resilience in handling increased gait variance through robust preprocessing using the autocorrelation-based filter.

**Keystroke modality:**

- Device diversity: Validated across desktops, laptops, and mobile devices, ensuring the algorithm accounts for variations in key layouts and typing ergonomics. Accuracy remained steady at 97.8%, with minimal deviations caused by keyboard size and user typing posture.
- Typing behavior: Adapted to fluctuating typing speeds, including burst typing and sporadic key presses, through normalization techniques in the keystroke scoring algorithm.

**Combined performance:** During transitions between stationary typing sessions and mobile walking scenarios, the system leveraged context-driven weight adjustment to emphasize the most reliable biometric signal for the given condition.

Dynamic Fusion maintained authentication accuracy above 97%, highlighting its ability to seamlessly adapt to the user’s activity and surroundings.

#### Experimental validation of dynamic weights

The dynamic weight adjustment mechanism underwent extensive testing to benchmark its efficacy against traditional static weight configurations. The validation process involved diverse experimental setups, user behaviors, and environmental conditions.

**Test configurations:**

- Static weights: A fixed weight distribution was applied (e.g.,$w_k$=0.5,$w_g$=0.5), irrespective of context. Achieved an accuracy of 94.8%, with increased susceptibility to FAR and FRR due to an inability to adapt to real-time variations.
- Dynamic Weights: Real-time adjustment of $w_k$ and $w_g$ based on contextual factors like typing frequency and gait variance. To rigorously evaluate the dynamic weight adjustment mechanism, experiments compared static and dynamic weight configurations. The performance metrics are summarized in Table 1, which highlights the improvements achieved using dynamic weights. Dynamic weights led to a significant increase in accuracy (from 94.8% to 98.25%) and a marked reduction in the False Accept Rate (FAR) and False Reject Rate (FRR), improving system reliability. These results underline the system’s ability to dynamically adapt biometric fusion based on user activity and environmental conditions.
- Context-Driven Benefits: The system demonstrated the ability to prioritize typing-based authentication during periods of high keyboard activity and shift focus to gait biometrics when user mobility was detected. This adaptability enhanced usability by minimizing false rejections during genuine user activity, even under challenging conditions like inconsistent gait cycles or erratic typing behavior.
- Comparative Analysis: The inclusion of dynamic weights improved authentication accuracy by 3.45% and significantly reduced the error rates. These results validate the importance of tailoring the fusion algorithm to contextual inputs for achieving reliable and seamless authentication.

**Table 1:** Performance metrics.

Configuration	Accuracy (%)	FAR (%)	FRR (%)
Static Weights	94.8	4.6	5.2
Dynamic Weights	98.25	2.1	2.35

### Comparative analysis of geature extraction methods

Table 2 presents the performance metrics of various feature extraction methods employed within the CMBSA for keystroke and gait biometrics. This section provides an in-depth analysis of these methods and contextualizes their impact on system performance.

- Keystroke biometrics: Wavelet Transform Filtering (WTF) employs multi-resolution analysis to isolate high-frequency noise from low-frequency keystroke features such as dwell time, flight time, and latency. Unlike FFT, which primarily operates in the frequency domain and can lose temporal resolution, WTF captures both time and frequency information. This dual advantage is critical for keystroke dynamics, as temporal patterns play a key role in user identification. For example^38^, utilized FFT for keystroke authentication, achieving a 95.2% accuracy but encountering higher False Reject Rates (FRR) due to noise from distractions or fatigue. WTF, integrated within our system, mitigated such challenges, delivering a 98.25% accuracy with a 2.5% Equal Error Rate (EER). This improvement stems from WTF’s ability to denoise typing data effectively while preserving essential keystroke features.
- Gait biometrics: Autocorrelation Filtering (ACF) is designed to detect periodic patterns and remove irregularities in gait cycles. Its efficacy surpasses that of Kalman Filtering, which, while adept at smoothing sensor data, often loses subtle periodic features crucial for biometric authentication. The study^39^ applied Kalman Filtering for wrist-based gait authentication, achieving a 96.4% accuracy with a 3.9% EER. By comparison, our system’s use of ACF reduced these errors, yielding a 2.5% EER and enhancing gait segment detection accuracy. ACF’s ability to retain periodic gait features while compensating for sensor inconsistencies underscores its suitability for biometric applications.
- Integration within CMBSA: The integration of WTF and ACF into the CMBSA framework elevates the system’s adaptability and robustness. Unlike traditional methods like PCA or Kalman Filtering, which require manual tuning to adapt to user behavior, CMBSA leverages dynamic scoring to ensure seamless scalability across real-world scenarios.

**Table 2:** Comparative performance of feature extraction methods for continuous multi-biometric systems.

Method	Accuracy (%)	EER (%)	FRR (%)	FAR (%)	Usability
CMBSA (WTF + ACF)	98.25	2.5	2.5	2.5	High (low latency, real-time)
FFT + Kalman Filtering	96.3	3.6	3.7	3.5	Medium (sensitive to noise and artifacts)
PCA	94.7	4.1	4.2	4.0	Low (requires manual tuning)
ICA	95.5	3.9	4.0	3.8	Medium (complex pre-processing)

### Impact of segment length and training set size

Segment Length: Using segments with fewer than four gait cycles led to a notable decline in performance, particularly for wrist-based authentication. Conversely, increasing the number of cycles beyond four did not yield significant improvements, suggesting that a four-cycle segment strikes the optimal balance between accuracy and real-time authentication needs.

Training Set Size: For both gait and keystroke dynamics, reducing the number of instances from the training set resulted in only minor increases in EER. For gait authentication, reducing from 45 to 10 instances had $a \le 1\%$ increase in EER, confirming the feasibility of deploying the system on devices with limited memory. Similarly, in keystroke dynamics, shorter typing samples led to increased EER due to insufficient data, while longer samples improved accuracy.

### System performance and discussion

The integration of keystroke and gait biometrics into a multimodal authentication system significantly enhances the overall performance. The fusion system benefits from the complementary strengths of both modalities:

- Keystroke authentication: Provides consistent verification based on typing patterns, reinforced by Wavelet Transform Filtering.
- Gait Authentication: Offers high accuracy through autocorrelation techniques, effectively detecting gait patterns even with variations.

The multimodal approach, combining these techniques, achieves an estimated combined accuracy of 96.4%. This system reduces individual biometric weaknesses, providing a more reliable and resilient authentication solution. The experimental results for the Context-Driven Multi-Biometric Scoring Algorithm (CMBSA) are summarized in Table 3, showcasing performance across 10 users with varying keystroke dynamics and gait biometrics. The keystroke scores, ranging from 0.65 to 0.90, and gait scores, from 0.68 to 0.86, reflect the system’s ability to accurately match current patterns to stored templates. The combined scores, which range from 0.66 to 0.88, demonstrate the effectiveness of the weighted fusion approach in integrating both biometric modalities.

Authentication decisions were made based on these combined scores, with lower scores resulting in rejections and higher scores leading to acceptance. The False Reject Rate (FRR) varied between 1.0% and 3.2%, while the False Accept Rate (FAR) ranged from 0.3% to 1.1%, and the Equal Error Rate (EER) was between 0.8% and 1.9%, indicating a balance in performance. Contextual factors, such as server score (0.7 to 0.9), time of day (from 08:00 to 23:00), and system configuration (High, Medium, Low), significantly influenced the accuracy and reliability of the biometric authentication process. The results underscore the CMBSA’s robustness and adaptability, highlighting its capacity to deliver reliable authentication in varied real-world scenarios.

**Table 3:** Experimental results for context aware multi-biometric scoring algorithm.

User ID	Keystroke Score	Gait Score	Combined Score	Authentication Decision	FRR	FAR	EER	Server Score	Time of Day	System Configuration
1	0.90	0.85	0.88	Accepted	1.5%	0.5%	1.0%	0.9	14:30	High
2	0.80	0.78	0.79	Accepted	2.0%	1.0%	1.5%	0.8	09:00	Medium
3	0.70	0.75	0.72	Rejected	3.0%	0.7%	1.8%	0.7	22:00	Low
4	0.85	0.82	0.83	Accepted	1.0%	0.3%	0.8%	0.85	12:00	High
5	0.65	0.68	0.66	Rejected	2.5%	0.9%	1.7%	0.75	16:00	Medium
6	0.78	0.80	0.79	Accepted	1.8%	0.6%	1.2%	0.8	10:00	High
7	0.90	0.85	0.88	Accepted	1.2%	0.4%	0.8%	0.9	18:00	High
8	0.72	0.70	0.71	Rejected	3.2%	1.1%	1.9%	0.7	23:00	Low
9	0.88	0.86	0.87	Accepted	1.3%	0.3%	0.8%	0.85	08:00	High
10	0.77	0.74	0.76	Rejected	2.7%	0.8%	1.7%	0.78	20:00	Medium

**Table 4:** Comparison of continuous authentication approaches.

Study	Modality	Method/Model	Accuracy	EER (%)	FAR (%)	FRR (%)	Key Findings	Usability Metric
CMBSA	Multimodal (Keystroke + Gait)	WTF + ACF	98.25%	2.35	2.5	2.5	High accuracy and usability, combining keystroke and gait biometrics using a dynamic scoring algorithm.	High
^28^	Keystroke Dynamics	Transformer-based Neural Network	95.2%	2.12	3.3	2.8	Real-time keystroke authentication using Transformer architecture.	Medium
^30^	Gait Biometrics (wrist)	LSTM + GRU Hybrid Model	96.8%	3.7	2.5	2.0	Enhances temporal gait feature extraction for continuous authentication.	Medium
^33^	Multimodal (Keystroke + Gait)	Deep Fusion Network	97.9%	3.4	1.8	1.9	Gait and keystroke fusion with deep learning reduces spoofing attempts.	High
^34^	Multimodal (Keystroke + Voice)	CNN + Autoencoder	97.3%	2.9	2.0	1.9	Combines keystroke and voice features, resisting impersonation attacks.	Medium
^31^	Gait Biometrics (smartphone)	Siamese Neural Networks	94.7%	4.1	3.2	2.9	Smartphone-based gait authentication with some sensor inconsistencies.	Low
^35^	Multimodal (Gait + Face)	CNN + Blockchain	99.8%	1.4	N/A	N/A	Blockchain integration for continuous gait and face authentication security.	High
^29^	Multimodal (Keystroke + Mouse)	Random Forest + SVM	97.5%	2.8	2.3	2.1	Keystroke and mouse biometrics achieve high accuracy for continuous monitoring.	Medium
^32^	Gait Biometrics (wrist)	BiLSTM + Autoencoder	96.4%	3.9	2.7	2.5	Enhances wrist-based gait dynamics for low-resource continuous authentication.	Medium
^36^	Multimodal (Keystroke + Gait)	Ensemble of CNN + LSTM	98.1%	2.6	2.2	1.9	Combines keystroke and gait biometrics for seamless continuous authentication.	High

The proposed method integrates Wavelet Transform Filtering (WTF) for keystroke dynamics and autocorrelation-based filtering (ACF) for gait biometrics to enhance the performance of continuous user authentication systems. Specifically, WTF is used for processing keystroke dynamics, addressing the noise inherent in typing patterns caused by distractions, fatigue, or minor errors. WTF leverages multi-resolution analysis, enabling the separation of high-frequency noise from low-frequency features, ensuring the capture of essential keystroke metrics such as dwell time, flight time, and latency. By denoising the raw data, WTF improves the quality and reliability of extracted features. For gait biometrics, ACF is applied to gait data, focusing on identifying periodic patterns and removing irregularities within gait cycles. This approach enables robust detection of continuous gait segments, improving the precision and reliability of gait-based authentication. These pre-processing methods contribute to the system’s high accuracy of 98.25% and an Equal Error Rate (EER) of 2.5% as shown in Table 4.

The usability of the system is also notably enhanced by these filtering methods. WTF reduces false negatives in keystroke dynamics, ensuring that legitimate users are not mistakenly rejected due to minor typing anomalies. Similarly, ACF ensures that gait cycles are correctly identified even with subtle variations in walking patterns. Both filtering techniques enable low-latency, real-time processing, which is crucial for seamless user authentication. The integration of Wavelet Transform Filtering and autocorrelation-based gait filtering with the Context-Driven Multi-Biometric Scoring Algorithm (CMBSA) creates a robust and flexible authentication system capable of adapting to evolving user behaviors over time, enhancing long-term effectiveness in diverse environments.

The novelty of this work lies in the synergy between WTF for keystroke dynamics, ACF for gait biometrics, and the CMBSA dynamic scoring approach. Traditional keystroke authentication systems typically rely on simpler filtering methods or may not address the noise effectively, while gait recognition often struggles with temporal inconsistencies. By combining these advanced techniques, the proposed method offers a more scalable and robust solution for continuous user authentication, ensuring seamless integration across a variety of real-world scenarios. This approach addresses both keystroke and gait-specific challenges, contributing to improved usability and reduced authentication errors.

### Robustness evaluation under noisy conditions

In real-world deployments, biometric signals are often subjected to noise due to sensor inaccuracies, environmental changes, and inconsistent user behavior. Although the BB-MAS dataset used in this study is preprocessed, we performed robustness testing by artificially introducing synthetic noise into both keystroke and gait data streams to simulate real-world interferences.

Three noise scenarios were created:

- **Mild Noise (N1):** Low-variance Gaussian noise added to keystroke timing and gait acceleration to simulate minor sensor jitter.
- **Moderate Noise (N2):** Combination of temporal jitter and simulated packet loss to mimic intermittent sensing errors or motion blur.
- **Severe Noise (N3):** Strong amplitude and timing distortions reflecting poor device positioning or noisy environments (e.g., crowded locomotion or unstable typing surfaces).

The system was evaluated under these noise conditions without re-training, using the same fusion logic. The results, shown in Table 5, demonstrate that the system maintains strong performance even under high-noise scenarios, highlighting the resilience of the CMBSA fusion approach.

**Table 5:** Robustness evaluation of CMBSA under varying noise conditions.

Condition	Accuracy (%)	EER (%)	FAR (%)	FRR (%)
Clean (No Noise)	98.25	2.35	2.5	2.5
Mild Noise (N1)	96.80	2.90	3.0	3.2
Moderate Noise (N2)	95.40	3.50	3.7	3.6
Severe Noise (N3)	93.90	4.10	4.3	4.2

These results confirm that the adaptive modality weighting and fallback strategies implemented in CMBSA allow the system to sustain secure, continuous authentication across noisy or degraded input conditions.

### Implementation and practical considerations

The proposed CMBSA-based continuous authentication framework is architected for real-time deployment with minimal computational overhead. To support responsiveness and seamless user experience, the system processes biometric data in short sliding windows (typically 3–5 seconds) and updates authentication decisions every 8–10 seconds, depending on user activity. The keystroke dynamics module performs lightweight feature extraction, including dwell time, flight time, and digraph latency computations, followed by Wavelet Transform Filtering (WTF) to denoise signal patterns. These operations are computationally inexpensive and complete within a few milliseconds on commodity hardware.

The gait analysis component uses triaxial accelerometer data, which is segmented and refined using autocorrelation-based filtering, achieving a time complexity of $O(n \log n)$ per window. Since the data windows are small and fixed in size, the processing remains real-time even on mobile devices. Fusion and decision-making are performed by CMBSA, which relies solely on basic arithmetic operations such as threshold comparison, linear scoring, normalization, and weight recalibration. The entire fusion pipeline is stateless, lightweight, and incurs negligible latency (complexity $O(1)$).

No deep learning inference is involved in the runtime decision loop, which eliminates the need for GPU acceleration or cloud-based computation. As a result, the system can run entirely on-device, preserving both latency and privacy. Benchmark tests indicate that the total end-to-end processing time per authentication cycle remains under 150–200 milliseconds on standard smartphones and laptops, satisfying practical real-time constraints. Overall, the proposed framework ensures efficient, unobtrusive authentication while remaining computationally scalable for deployment in real-world mobile, desktop, and edge-computing environments.

#### Real-world deployment scenarios

The fusion of keystroke dynamics and gait biometrics in the proposed system is especially effective for a wide range of real-world use cases, where continuous, seamless, and non-intrusive user authentication is essential. By leveraging the complementary availability of keystroke and gait signals across different contexts, the system ensures robust coverage in both static and dynamic environments. Below are key application domains where this approach is particularly beneficial:

- **Enterprise and remote work environments:** Users working from home or office frequently alternate between typing at a desk and moving around. The proposed system maintains authentication seamlessly by prioritizing keystroke dynamics during desk work and shifting to gait recognition when the user moves within the premises, preventing session hijacking or unauthorized access in shared environments.
- **Mobile banking and financial services:** Continuous authentication is critical in mobile applications dealing with sensitive financial data. The system can passively monitor touchscreen keystroke inputs and gait patterns while the user interacts with a smartphone, enabling high security without requiring repeated logins or user interruptions.
- **Healthcare and mHealth monitoring:** In mobile health (mHealth) applications, secure and continuous authentication is needed for accessing personal health data. The fusion of gait and keystroke biometrics ensures low-effort and passive verification while patients or healthcare professionals interact with mobile or wearable devices.
- **Educational platforms and online examination systems:** For secure e-learning or remote examination platforms, keystroke dynamics can authenticate users during typing-based interactions (e.g., answering questions), while gait patterns can be used to detect if the user leaves the device area or attempts impersonation.
- **Military and security-critical systems:** In high-security environments where continuous identity verification is critical, the fusion strategy ensures persistent monitoring across various user states. Keystroke data secures terminal interactions, while gait ensures authentication continuity when personnel move between zones or terminals.
- **Public terminals and shared devices:** In settings such as libraries, kiosks, or co-working spaces, the system can maintain a continuous trust score even when one modality (e.g., gait) is unavailable, using the adaptive fallback strategy based on context-aware scoring, thereby avoiding abrupt logouts or intrusive re-verifications.

By supporting passive data collection and adaptive fusion, the proposed system offers a high degree of flexibility and resilience to signal dropout, making it highly suitable for ubiquitous computing environments that demand real-time, context-aware security.

### Privacy considerations and data protection

Continuous monitoring of behavioral biometrics such as keystroke dynamics and gait data inherently introduces privacy concerns, particularly regarding data sensitivity, user consent, and storage practices. To address these challenges, the proposed system has been architected with privacy-by-design principles. All biometric data processing including signal acquisition, feature extraction, score computation, and adaptive fusion is executed locally on the user’s device, eliminating the need to transmit or store raw biometric data on external servers. This reduces the risk of data interception or centralized breaches. Furthermore, no identifiable raw input (e.g., exact keystrokes or full motion traces) is retained; only non-reversible statistical scores are used in authentication decisions. In practical deployments, the system can be integrated with user-centric consent mechanisms, allowing users to opt in, review collected data types, and revoke permissions at any time. Future work will explore decentralized on-device processing and advanced consent mechanisms to enhance data privacy without compromising usability.

### Limitations

The approach presented has several limitations that need consideration. Environmental and contextual factors can impact the performance of both keystroke dynamics and gait biometrics. For instance, variations in typing conditions or walking surfaces may affect the accuracy of the system. Device dependency is another challenge, with wrist-worn accelerometers potentially suffering from interference due to arm swing, while pocket-worn devices generally perform better but require consistent user adherence. The system’s ability to adapt to significant changes in user behavior, such as alterations in typing or walking patterns, can also be limited, necessitating continuous recalibration. Additionally, the computational complexity of the Context-Driven Multi-Biometric Scoring Algorithm (CMBSA) may increase the system’s load, affecting real-time performance. Privacy concerns arise from the continuous monitoring of user behavior, requiring stringent data security measures and transparency. Lastly, the system’s performance, validated with a specific user set, may not generalize well to other groups or contexts, highlighting the need for broader evaluation.

### Usability analysis

The usability of the multi-modal authentication system, which integrates keystroke dynamics and gait biometrics, is a key consideration in evaluating its practical effectiveness. This approach offers several usability benefits.

Benefits:

- Seamless integration: The multi-modal system is designed to operate seamlessly in the background, requiring minimal user interaction once set up. The continuous authentication provided by keystroke dynamics and gait biometrics ensures that users are authenticated in real-time without the need for explicit login actions, enhancing user convenience and reducing interruptions.
- Adaptive Functionality: The Context-Driven Multi-Biometric Scoring Algorithm (CMBSA) dynamically adjusts the weight of each biometric modality based on contextual factors such as user activity and environment. This adaptability allows the system to optimize authentication accuracy and performance, even as user behavior or conditions change.
- Enhanced security: By combining multiple biometric modalities, the system provides a higher level of security compared to single-modality approaches. This layered authentication method reduces the likelihood of unauthorized access and improves overall system reliability.
- Reduced false rejections and acceptances: The fusion of keystroke and gait data enhances the system’s ability to distinguish between genuine users and imposters, thereby reducing both false reject rates (FRR) and false accept rates (FAR). This results in a more reliable and user-friendly authentication experience.

## Conclusion

In this study, the effectiveness of integrating keystroke dynamics and gait biometrics into a continuous user authentication system is demonstrated. The evaluation of individual and combined modalities highlights several key insights. The keystroke dynamics module, processed through advanced Wavelet Transform Filtering (WTF), achieved an impressive authentication accuracy of approximately 97%. This high accuracy was corroborated by ROC curve analysis, which indicated strong performance in distinguishing between genuine and imposter users. Similarly, the gait-based authentication module, utilizing triaxial accelerometers and an Autocorrelation (AC) Filter, achieved notable results with an Equal Error Rate (EER) of 2.5% for wrist-based devices and an AUC of 99.5%.

The fusion of these modalities, facilitated by the Context-Driven Multi-Biometric Scoring Algorithm (CMBSA), resulted in a combined accuracy of approximately 98.25%. This integration not only enhanced overall accuracy but also significantly reduced False Accept Rates (FAR) and False Reject Rates (FRR), thereby providing a more secure and reliable authentication solution. Importantly, the multi-modal approach improves usability by adapting to user behavior and context, ensuring a seamless authentication experience across different scenarios.

The results underline the strength of combining keystroke and gait dynamics to create a robust continuous authentication system. In response to recent literature addressing the cyclostationarity of biometric signals, future studies are encouraged to investigate whether keystroke dynamics and gait signals exhibit cyclostationary behavior. Incorporating spectral correlation functions (SCF) in signal analysis may offer a more robust alternative to traditional autocorrelation-based filtering methods. A comparative study between SCF and standard correlation techniques could further enhance the accuracy and resilience of biometric systems.

This research contributes to the advancement of secure, adaptive, and user-friendly authentication mechanisms and lays the foundation for future innovations in continuous user authentication using behavioral biometrics.

## Data Availability

The BB-MAS dataset used in this study is publicly available and can be accessed at:https://paperswithcode.com/dataset/bb-mas. All experimental results are based on this dataset. Any additional data or scripts used for analysis are available from the corresponding author upon reasonable request.
